# Genes differentially expressed between pathogenic and non-pathogenic *Entamoeba histolytica* clones influence pathogenicity-associated phenotypes by multiple mechanisms

**DOI:** 10.1371/journal.ppat.1011745

**Published:** 2023-12-22

**Authors:** Juliett Anders, Constantin König, Corinna Lender, Arne Hellhund, Sarah Nehls, Ibrahim Shalabi, Barbara Honecker, Stephan Lorenzen, Martin Meyer, Jenny Matthiesen, Dániel Cadar, Thomas Roeder, Nahla Galal Metwally, Hannelore Lotter, Iris Bruchhaus

**Affiliations:** 1 RG-Host Parasite Interaction, Bernhard Nocht Institute for Tropical Medicine, Hamburg, Germany; 2 Department of Infectious Disease Epidemiology, Bernhard Nocht Institute for Tropical Medicine, Hamburg, Germany; 3 Department of Arbovirology and Entomology, Bernhard Nocht Institute for Tropical Medicine, Hamburg, Germany; 4 Kiel University, Department Molecular Physiology, Zoology, Kiel, Germany; 5 DZL, German Center for Lung Research, ARCN, Airway Research Center North, Kiel, Germany; 6 RG Molecular Infection Immunology, Bernhard Nocht Institute for Tropical Medicine, Hamburg, Germany; 7 Biology Department, University of Hamburg, Hamburg, Germany; University of Geneva Faculty of Medicine: Universite de Geneve Faculte de Medecine, SWITZERLAND

## Abstract

Recently, two genes involved in amoebic liver abscess formation in a mouse model were identified by their differential expression of non-pathogenic (A1^np^) and pathogenic (B2^p^) clones of the *Entamoeba histolytica* isolate HM:1-IMSS. While overexpression of a gene encoding the metallopeptidase EhMP8-2 reduces the virulence of the pathogenic clone B2^p^, overexpression of the gene *ehi_127670* (*ehhp127*), encoding a hypothetical protein, increases the virulence of the non-pathogenic clone A1^np^, while silencing this gene in the pathogenic B2^p^ reduces virulence. To understand the role of both molecules in determining the pathogenicity of *E*. *histolytica*, silencing, and overexpression transfectants were characterized in detail. Silencing of *ehmp8-2*, of the homologous gene *ehmp8-1*, or both in non-pathogenic A1^np^ trophozoites significantly altered the transcript levels of 347, 216, and 58 genes, respectively. This strong change in the expression profiles caused by the silencing of *ehmp8-1* and *ehmp8-2* implies that these peptidases regulate the expression of numerous genes. Consequently, numerous phenotypic characteristics, including cytopathic, hemolytic, and cysteine peptidase activity, were altered in response to their silencing. Silencing of *ehhp127* in pathogenic B2^p^ trophozoites did not affect the expression of other genes, whereas its overexpression in non-pathogenic A1^np^ trophozoites results in an altered expression of approximately 140 genes. EhHP127 is important for trophozoite motility, as its silencing reduces, while its overexpression enhances movement activity. Interestingly, the specific silencing of *ehhp127* also significantly affects cytopathic, cysteine peptidase, and hemolytic activities. All three molecules characterized in this study, namely EhMP8-1, EhMP8-2, and EhHP127, are present in amoeba vesicles. The results show that *ehmp8-2* and *ehhp127* are not only differentially expressed between pathogenic and non-pathogenic amoebae, but that they also significantly affect amoeba pathogenicity-associated phenotypes by completely different mechanisms. This observation suggests that the regulation of amoeba pathogenicity is achieved by a complex network of molecular mechanisms rather than by single factors.

## Author summary

The human pathogen *Entamoeba histolytica* can live asymptomatically in the intestine or become invasive and cause fatal liver abscesses. Approximately 15,000 people die each year as a result of an amoebic infection. Recently, two clones with different pathogenicity (A1^np^: non-pathogenic; B2^p^: pathogenic) derived from the *E*. *histolytica* isolate HM:1-IMSS were compared at transcriptome level. Two highly differentially expressed genes (*ehhp127* encoding a hypothetical protein and *ehmp8-2* encoding a metallopeptidase) were identified. Analysis of *E*. *histolytica* transfectants showed that silencing of *ehhp127* and overexpression of *ehmp8-2* in B2^p^ trophozoites reduced amoebic liver abscess formation in the mouse model. In this study, we characterized *E*. *histolytica* silencing and overexpression transfectants of *ehmp8-2*, as well as of the homologous gene *ehmp8-1* and of *ehhp127*. It was shown that the altered expression of the metallopeptidase genes has a strong influence on the expression of a large number of genes and that the phenotype is strongly altered as a result. Silencing of *ehhp127* does not affect the overall expression profile. However, specific silencing impairs motility and cytopathic activity. All three molecules have been shown to be localized in trophozoite vesicles.

## Introduction

*Entamoeba histolytica* is the causative agent of amoebiasis, which kills approximately 15,000 people annually [1]. *E*. *histolytica* is an intestinal protozoan that, for reasons that are not yet understood, can become invasive, penetrate the intestinal mucosa, invade the tissue, and migrate via the bloodstream to the liver. This invasion can lead to the development of amoebic colitis and the formation of amoebic liver abscesses (ALAs).

In order to identify virulence factors of *E*. *histolytica* involved in the formation of ALAs, in recent years we have analyzed amoeba clones (A1^np^ and B2^p^) originally derived from the same isolate (HM-1:IMSS) but differing in their ability to form ALAs [2–5]. Seven days after injection of amoebae into the liver of mice, no ALAs can be detected in animals infected with A1^np^ trophozoites, whereas ALAs are present in animals infected with B2^p^ trophozoites [4].

Analysis of the transcriptomes of the non-pathogenic clone A1^np^ and the pathogenic clone B2^p^ revealed 76 genes that are differentially expressed between the two clones. These include 46 genes that are significantly more highly expressed in A1^np^ trophozoites and 30 genes that are significantly more highly expressed in B2^p^ trophozoites (fold change > 3, *p*adj < 0.05) [4].

The second most highly differentially expressed gene in non-pathogenic A1^np^ trophozoites is *ehi_042870* (fold change 149), which encodes the cell surface protease gp63 (metallopeptidase EhMP8-2) [4]. The upregulation of *ehmp8-2* in pathogenic B2^p^ trophozoites leads to a loss of virulence of these transfectants, thus they are no longer able to form ALAs [4]. Silencing of *ehmp8-2* expression does not alter the non-pathogenic phenotype of A1^np^ [5]. Recently, the genome of *E*. *histolytica* was analyzed for peptidase-encoding genes. A total of 79 peptidase-encoding genes were identified. The largest group with 45 genes encodes cysteine peptidases (CPs). This is followed by metallopeptidase-encoding genes (21 members), serine peptidase-encoding genes (9 members) and aspartate peptidase-encoding genes (4 members) [6]. The 21-member group of metallopeptidases consists of 11 families, including two members of the M8 (leishmanolysin/gp63 like) family (EhMP8-1 (EHI_200230) and EhMP8-2 (EHI_042870)) [6]. Of all metallopeptidases, only EhMP8-1 has been characterized to date [7]. EhMP8-1 was detected in vesicles in all trophozoites examined. Furthermore, the protein was also detected on the cell surface in some of the trophozoites [7]. Silencing of *ehmp8-1* resulted in increased cytoadhesion to cell monolayers, decreased cytopathic activity and motility, and increased phagocytosis [7]. EhMP8-1 and EhMP8-2 contain the conserved HEXXH motif of the M8 family and the conserved C-terminal amino acids histidine and methionine [8]. EhMP8-1 consists of 643 amino acids, a putative signal peptide of 15 amino acids, and a putative transmembrane domain at position 605–627. EhMP8-2 consists of 662 amino acids, a putative signal peptide of 16 amino acids, and a putative transmembrane domain at position 598–620. Both metallopeptidases share only 32% sequence identity (49% similarity) (AmoebaDB, release 61, 15 Dec 2022). While *ehmp8-2* is differentially expressed between A1^np^ and B2^p^, as mentioned above, this is not the case for *ehmp8-1* [4]. Interestingly, there is only one member of the M8 family in the human non-pathogenic species *E*. *dispar*, which is 92% identical to the *E*. *histolytica* metallopeptidase EhMP8-2 [6]. It can therefore be speculated that lower virulence correlates with the presence or the expression of an *ehmp8-2* homologue.

The most differentially expressed gene (*ehi_127670*) between the pathogenic clone B2^p^ and the non-pathogenic clone A1^np^ encodes a hypothetical protein (EhHP127) and is 193-fold higher expressed in pathogenic B2^p^ trophozoites [4]. Pathogenic B2^p^ trophozoites, in which the expression of *ehhp127* is silenced, were significantly impaired in their ability to induce ALA formation in mice [5]. Overexpression of *ehhp127* in the non-pathogenic A1^np^ trophozoites also increases the pathogenicity of these clones as 4 out of 9 infected animals developed abscesses, which was nevertheless not significant [4].

Increased expression of *ehi_127670* (16.1-fold) was also shown in the isolate G3 (*amoebapore*-silenced) compared to the pathogenic HM-1:IMSS isolate (**primary contact:** Carol Gilchrist, University of Virginia, School of Medicine; **source version:** 2011-10-06; **release # / date:** AmoebaDB rel. 1.0, 2005-JAN-01), which shows a hierarchy of pathogenicity factors with amoebapore being essential. In addition, increased expression of *ehhp127* was detected after the adaptation of *E*. *histolytica* to 2 μM auranofin. However, this adaptation leads to the regulation of the expression of several hundred genes, suggesting that it is a very complex adaptation mechanism. Auranofin is an antirheumatic drug that targets the mammalian thioredoxin reductase (TrxR), but is also highly effective against a variety of pathogenic bacteria and protozoan parasites [9]. Recently, a study analyzed the expression profile of *E*. *histolytica* isolated from the clinical specimens of three patients. Expression of *ehhp127* was detected in isolate Ax19 from an ALA patient, whereas no expression was detected in isolate Ax11 from an asymptomatic carrier and in isolate Ax22 from a patient with amoebic colitis [10].

In this study, we aim to understand the functions of EhMP8-1, EhMP8-2, and EhHP127 using silencing and overexpression transfectants and to determine their relevance for virulence development using various *in vitro* assays. Our results show that silencing of *ehmp8-1* and/or *ehmp8-2* in A1^np^ trophozoites as well as overexpression of *ehhp127* in A1^np^ trophozoites affects the expression of a large number of genes, whereas silencing of *ehhp127* in B2^p^ trophozoites has no effect on the expression profile of other genes. It is therefore not surprising that silencing of metallopeptidase-encoding genes affects several phenotypic characteristics of amoebae, such as cytopathic, cysteine peptidase and hemolytic activities. Motility is impaired in both overexpression and silencing EhHP127 transfectants, while silencing also has a negative effect on cytopathic, cysteine and hemolytic activity. The metallopeptidases and EhHP127 have in common that they can be detected in trophozoite vesicles.

In conclusion, EhHP127 and the metalloproteinase EhMP8-2 not only direct pathogenicity in opposite directions, but they also employ different mechanisms to induce different phenotypes associated with pathogenicity.

## Results

### Experimental manipulation of *ehmp8-1* and *ehmp8-2* expression alters the transcript profiles of pathogenic and non-pathogenic amoeba clones

Recently, *ehmp8-1* was shown to be expressed in the non-pathogenic clone A1^np^ at almost the same level as in the pathogenic clone B2^p^ (reads: 2028 vs 2228; [4]). The expression level of *ehmp8-2* in clone A1^np^ is only about half that of *ehmp8-1* (reads: 1098), while *ehmp8-2* is almost not expressed at all in clone B2^p^ (reads: 7.4) [4].

**Fig 1:**
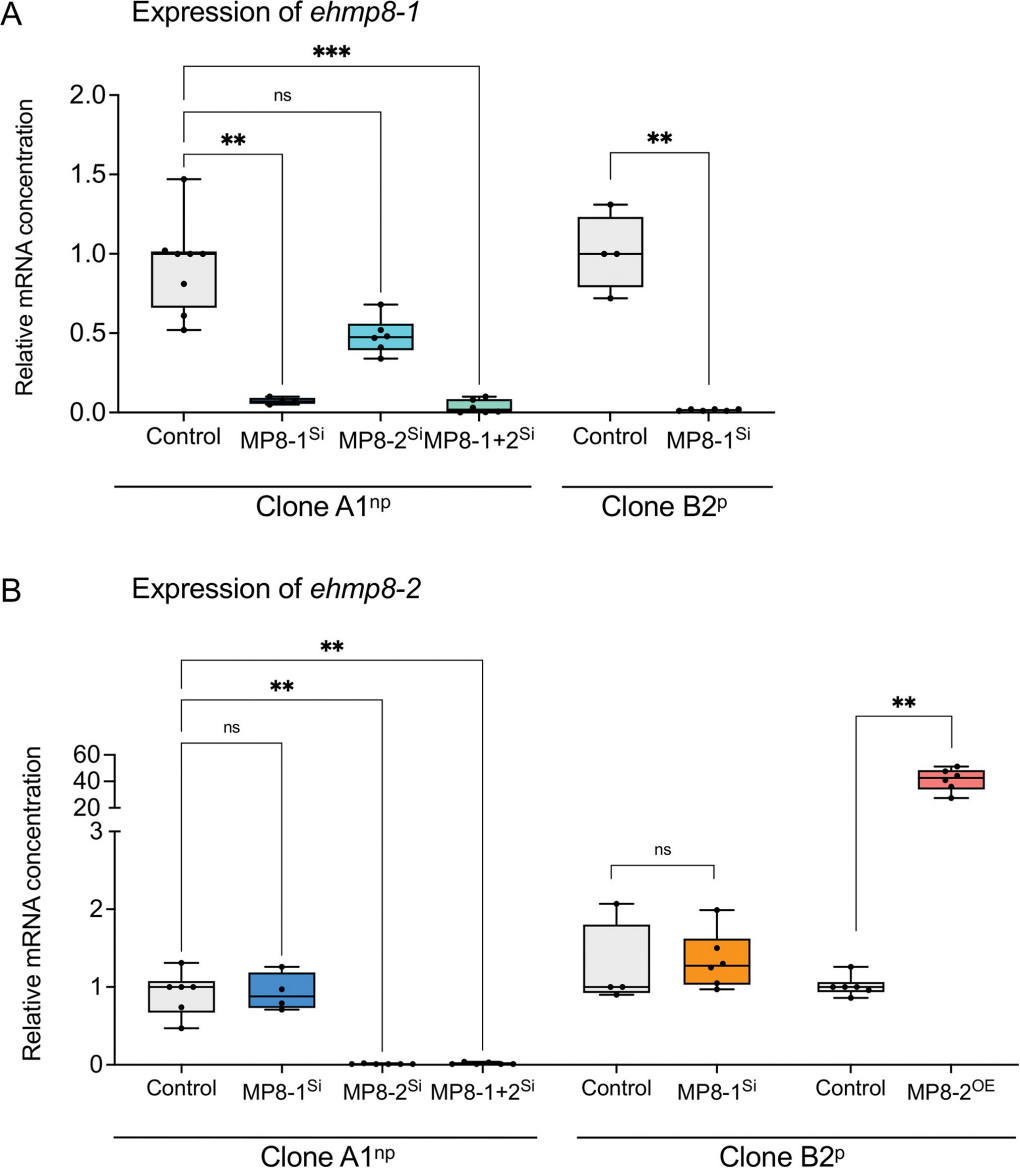
mRNA expression profile of *ehmp8-1* (A) and *ehmp8-2* (B) in A1^np^ and B2^p^ silencing and overexpression transfectants by means of RT-qPCR. RNA isolated from trophozoites was transcribed into cDNA and used for qPCR with SYBR Green to determine the relative mRNA concentration of *ehmp8-1* (A) and *ehmp8-2* (B) of the silencing transfectants A1^np^MP8-1^Si^, A1^np^MP8-2^Si^, A1^np^MP8-1+2^Si^, B^p^MP8-1^Si^ and the overexpression transfectant B2^p^MP8-2^OE^. Controls: Non-transfected A1^np^ or B2^p^ trophozoites. Actin was used as a calibrator and controls were normalized to 1. n = 2–4 (in duplicate). ns: not significant, **p < 0.01, ***p < 0.001 (non-parametric ANOVA test and Mann-Whitney *U* test).

To characterize the metallopeptidases, transfectants of the non-pathogenic clone A1^np^ were generated in which the expression of *ehmp8-1* (A1^np^MP8-1^Si^), *ehmp8-2* (A1^np^MP8-2^Si^) or both (A1^np^MP8-1+2^Si^) was silenced. Furthermore, transfectants of the pathogenic clone B2^p^ were generated in which *ehmp8-1* (B2^p^MP8-1^Si^) was silenced or *ehmp8-2* (B2^p^MP8-2^OE^) was overexpressed. Real-time quantitative PCR (qRT-PCR) confirmed the successful silencing and overexpression of the corresponding genes in the transfectants (Fig 1A and 1B and S1 Prism).

**Fig 2:**
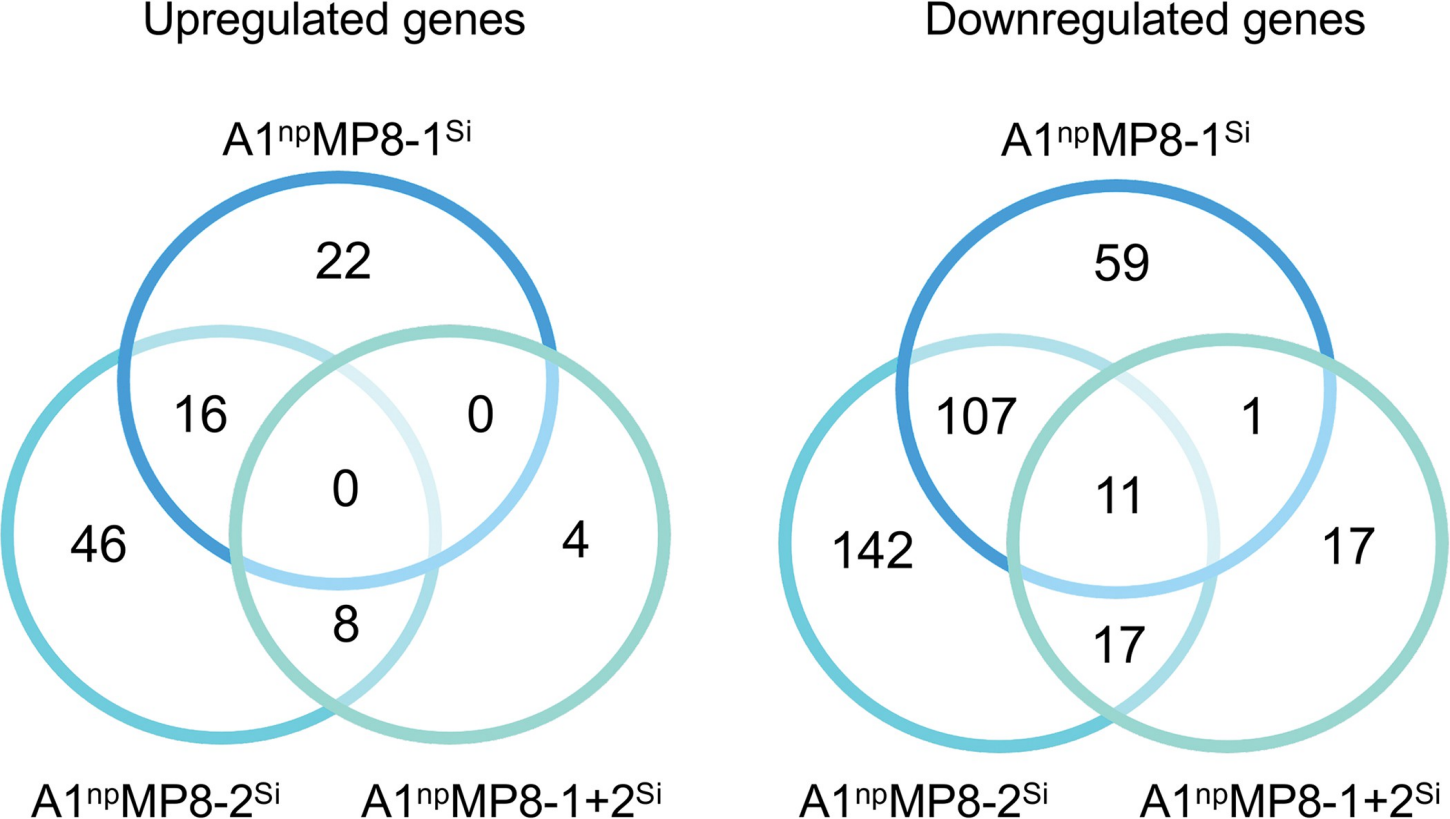
Venn diagram of differentially expressed genes in metallopeptidase silencing transfectants of non-pathogenic clone A1^np^ (A1^np^MP8-1^Si^, A1^np^MP8-2^Si^, A1^np^MP8-1+2^Si^). *P*adj < 0.05, fold change > 1.8.

**Fig 3:**
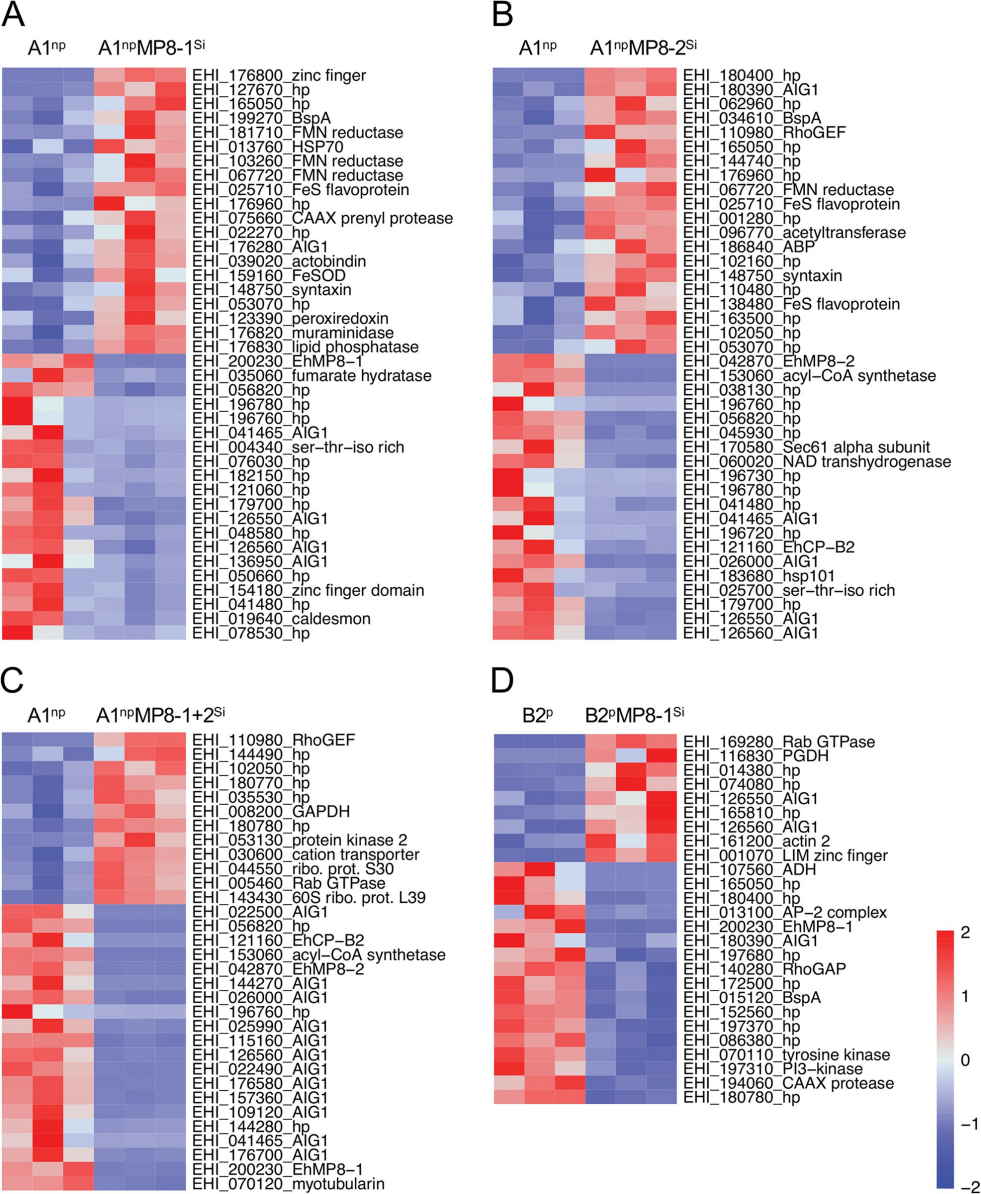
Heatmap of significantly differentially expressed genes (> 1.8 fold, *p*adj < 0.05) in the different *E*. *histolytica* transfectants silencing or overexpressing *ehmp8-1*, *ehmp8-2* or both compared to the corresponding controls. A maximum of 20 up- or downregulated genes with the highest fold change are shown. A. A1^np^ (control) versus A1^np^MP8-1^Si^, B. A1^np^ (control) versus A1^np^MP8-2^Si^, C. A1^np^ (control) versus A1^np^MP8-1+2^Si^, D. B2^p^ (control) versus B2^p^MP8-1^Si^.

**Fig 4:**
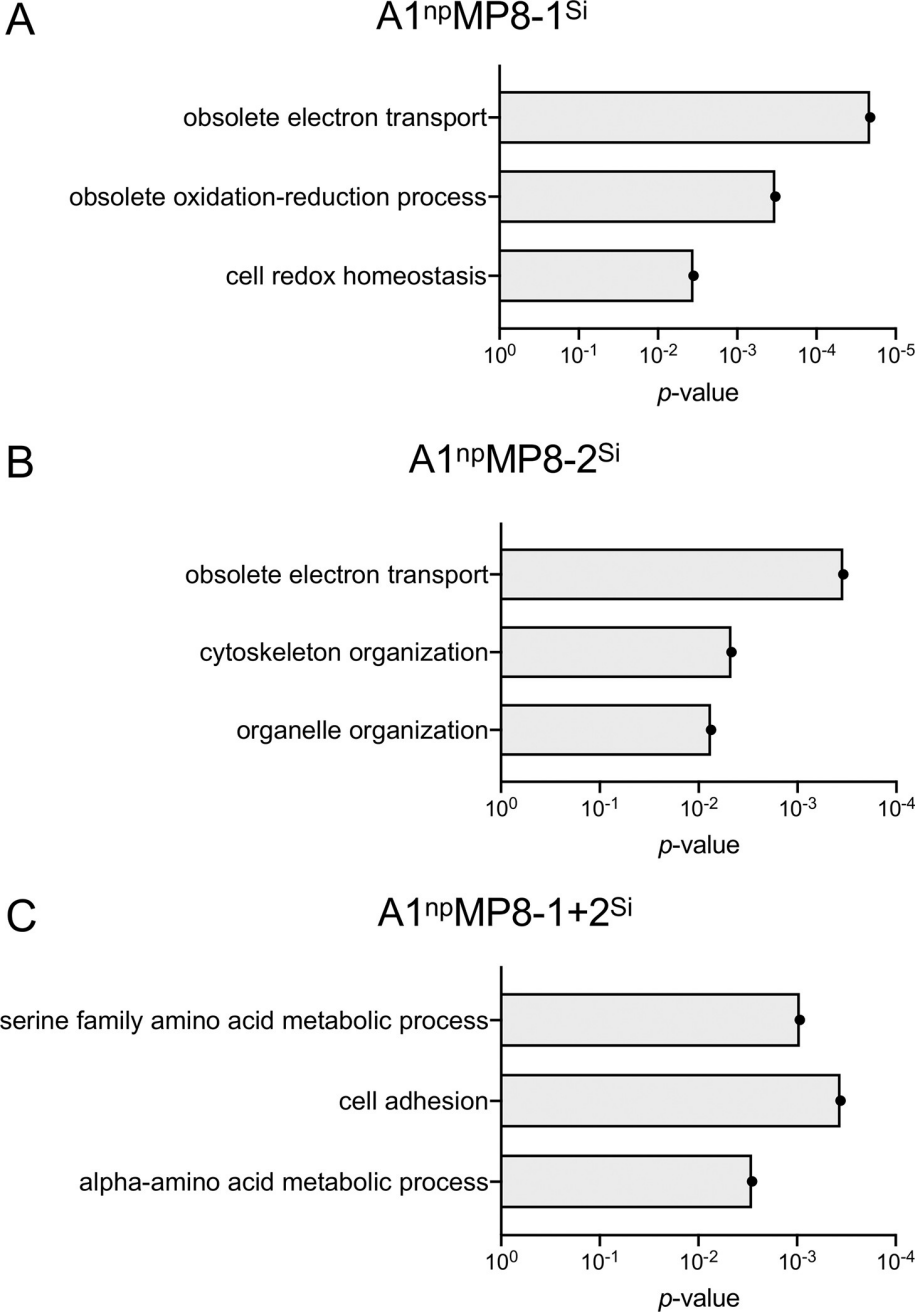
GO-BP analysis of differential expressed genes in (A) A1^np^MP8-1^Si^ transfectant, (B) A1_np_MP8-2^Si^ transfectant and (C) A1^np^MP8-1+2^Si^ transfectant.

Transcriptome analyses of the transfectants showed that silencing had a significant effect on the expression of other genes (*p*adj < 0.05, fold change > 1.8). Silencing of *ehmp8-1* in the non-pathogenic clone A1^np^ (A1^np^MP8-1^Si^ transfectant) resulted in altered expression of 216 genes (38 upregulated, 178 downregulated), silencing of *ehmp8-2* (A1^np^MP8-2^Si^ transfectant) resulted in altered expression of 347 genes (70 upregulated, 277 downregulated), and silencing of both metallopeptidase genes (A1^np^MP8-1+2^Si^ transfectant) resulted in altered expression of 58 genes (12 upregulated, 46 downregulated) (Figs 2, 3A–3C and S1 and S1–S3 Tables). Depending on the transfectant, between 3.8 and 4.7 times more genes were downregulated than upregulated. Gene ontology (GO) analyses revealed that silencing of *ehmp8-1* significantly affected five GO-biological processes (GO-BP) terms, including “obsolete electron transport” and “cell redox homeostasis” (*p* < 0.01) (Fig 4A and S4 Table). Within the GO term “cellular component” (GO-CC), there are only “plasma membrane” and “cell periphery” significantly affected (*p* < 0.01) (S4 Table). The GO term "molecular function” (GP-MF) contains nine significant terms, mainly involved in oxidoreductase and antioxidant activity (*p* < 0.01) (S4 Table).

Silencing of *ehmp8-2* has a significant influence on GO-BP terms “obsolete electron transport”, “cytoskeleton organization” and “organelle organization” (Fig 4B and S5 Table). Within GO-CC, only “chromosome” is significantly affected (*p* = 0.01), and GO-MF includes seven significant terms e.g., “FMN reductase activity”, “oxidoreductase activity, acting on the CH-NH group of donors” and “actin binding” (*p* < 0.01) (S5 Table). When both *ehmp8-1* and *ehmp8-2* are silenced, four GO-BP terms are significantly affected including “adhesion” and “amino acid metabolism” (Fig 4C and S6 Table). Within GO-CC no significantly regulated term was identified. For GO-MF an influence of twelve terms was observed, including”GTP binding” and “metallopeptidase activity” (S6 Table). However, it is important to note that about 47% of the differential expressed genes encode hypothetical proteins (S1–S3 Tables).

Looking at the genes individually, *ehhp127* was found to be the second most overexpressed gene in A1^np^EhMP8-1^Si^ transfectants (22-fold change, *p*adj 1,67E-19; Figs 3A and S1 and S1 Table). Furthermore, the expression of genes encoding proteins that protect against oxidative stress, such as peroxiredoxin, NADPH-dependent FMN reductase domain-containing protein, NADPH-dependent FMN reductase domain-containing protein, and iron-containing superoxide dismutase, are significantly upregulated. In addition, genes encoding the peptidases CAAX prenyl protease and EhCP-A7 are significantly upregulated whereas *ehcp-a4* is downregulated (Figs 3A and S1 and S1 Table). The 20 most downregulated genes also included four *aig* genes (between 5.3- and 6.7-fold) (Figs 3A and S1 and Tables 1 and S1).

**Table 1:** *Aig1* genes with significantly differential expression in A1^np^MP8-1^Si^, A1^np^MP8-2^Si^, A1^np^MP8-1+2^Si^, B2^p^MP8-1^Si^, and A1^np^HP127^OE^ transfectants compared to respective controls.

*Aig1* gene (Accession number)	A1^np^MP8-1^Si^		A1^np^MP8-2^Si^		A1^np^MP8-1+2^Si^		B2^p^MP8-1^Si^		A1^np^HP127^OE^	
	UP^1^	DOWN^2^	UP	DOWN	UP	DOWN	UP	DOWN	UP	DOWN
EHI_176280	X									X
EHI_136950		X		X						
EHI_126560		X		X			X			X
EHI_126550		X		X		X	X			X
EHI_041465		X								
EHI_180390			X							
EHI_026000				X						
EHI_025990				X						
EHI_176700				X		X				X
EHI_022490				X		X				
EHI_041465				X		X				
EHI_089670						X				X
EHI_157360						X				X
EHI_144270						X				
EHI_176590										X
EHI_176580										X
EHI_109120										X
EHI_144270										X

^1^Significant upregulation of expression ^2^Significant downregulation of expression (*p*adj < 0.05, fold change > 1.8).

The second most upregulated gene in the A1^np^EhMP8-2^Si^ transfectant (EHI_180390; fold change 8.7, *p*adj 4.9E-10) also encodes a protein of the AIG1 family (Figs 3B and S1 and Tables 1 and S2). However, a total of 8 genes encoding AIG1 family proteins are downregulated, four of them in the 20 most downregulated genes (Figs 3B and S1 and Tables 1 and S2). In addition, as in A1^np^EhMP8-1^Si^ transfectants, a number of genes that could encode for antioxidants (NADPH-dependent FMN reductase domain containing protein, iron-sulfur flavoprotein, iron hydrogenase, peroxiredoxin) are upregulated. It is also striking that many genes encoding proteins related to the cytoskeleton and surface proteins are downregulated (surface antigen ariel1, myotubularin, calponin homology domain protein, formin homology 2 family protein, caldesmon, actin-binding protein, Gal/GalNAc lectin 35 kDa subunit, Gal/GalNAc lectin 170 kDa subunit, filopodin, villin, formin homology 2 family protein, villidin, actinin-like protein) (Figs 3B and S1 and S2 Table).

Looking at the A1^np^EhMP8-1+2^Si^ silencing transfectants it was surprising that simultaneous silencing of both metallopeptidase genes had the least effect on the gene expression profile and that few genes were found in the intersection of all three silencing transfectants (Fig 2). However, it was very striking that 20 of the 48 downregulated genes belonged to the *aig1* family, twelve of which were among the 20 most highly regulated genes (Figs 3C and S1 and Tables 1 and S3). In addition, the genes encoding the large and small subunits of the Gal/GalNAc lectin and the cysteine peptidase EhCP-B2 were also downregulated (S1 Fig and S3 Table).

Silencing of *ehmp8-1* in the pathogenic clone B2^p^ regulates the expression of only a small number of genes. The expression of a total of 26 genes was significantly affected, of which 9 were upregulated and 17 were downregulated in their expression (Figs 3D and S1 and S7 Table). Again, two of the nine upregulated genes are *aig1* genes, while one *aig1* gene was downregulated (Tables 1 and S7 and Figs 3D and S1).

### Silencing and overexpression of metallopeptidase genes affect cytopathic, cysteine peptidase and hemolytic activities

**Fig 5:**
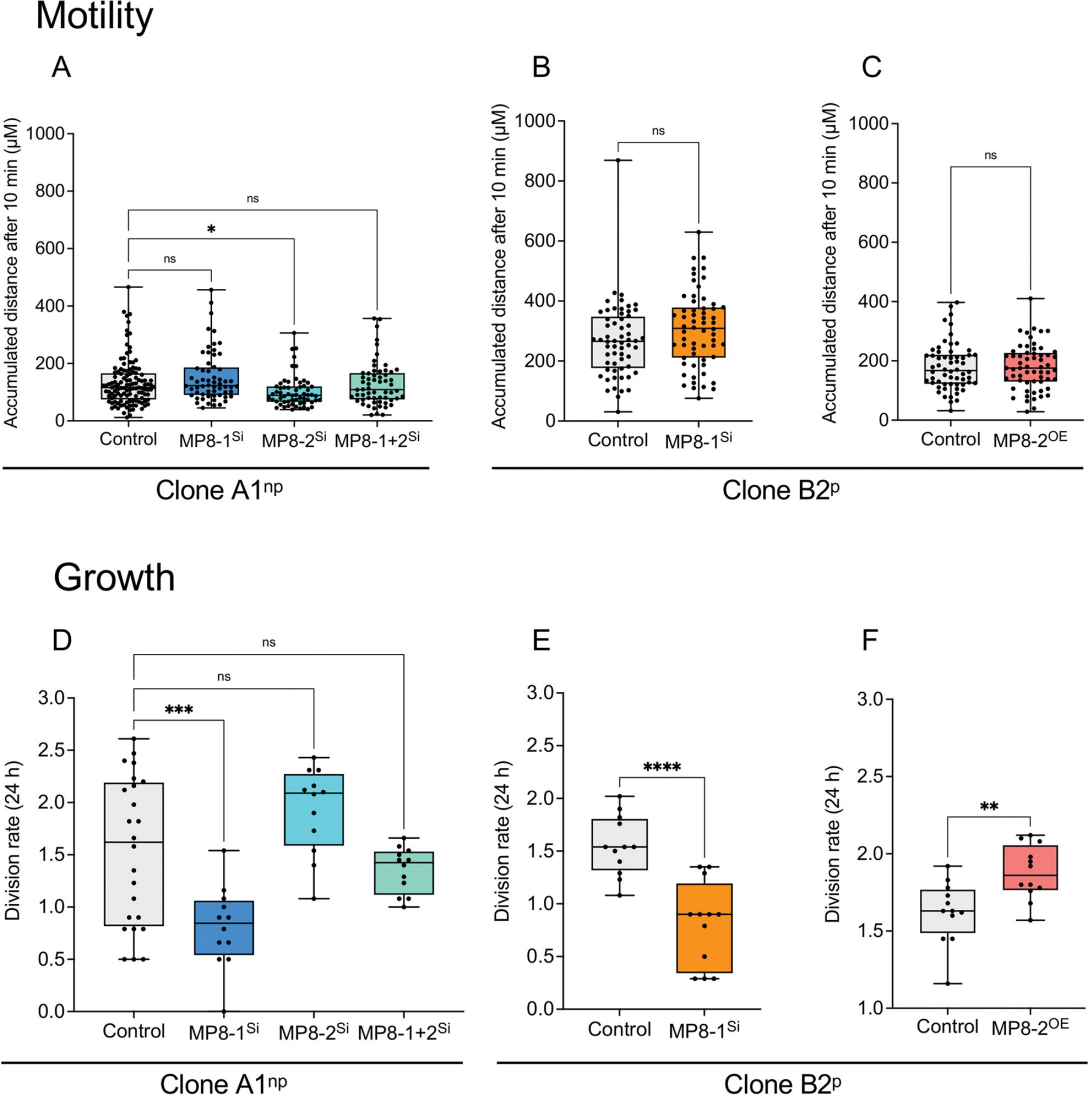
Determination of motility (A-C) and growth (D-F) of silencing transfectants A1^np^MP8-1^Si^, A1^np^MP8-2^Si^, A1^np^MP8-1+2^Si^ and B2^p^MP8-1^Si^ and overexpression transfectant B2^p^MP8-2^OE^. Non-transfected A1^np^ and B2^p^ trophozoites were used as controls for silencing transfectants and B2^p^ trophozoites transfected with the control plasmid pNC were used as control for overexpression transfectants. To determine motility (A-C), the accumulated distance (μm) was measured after 10 min. For each transfectant/control 60 amoebae were analysed. For each experiment, 3 biological replicates were used and the speed of movement was determined for 20 amoebae. To determine growth rate (D-F), 500 trophozoites of each clone were seeded into a 24-well plate, and the cells were counted every 24 h over 72 h. Experiments were performed four times in triplicate. Significance was determined using one-way ANOVA (A, D) and unpaired *t* test (B/C; E/F) (ns: not significant, *p < 0.05, **p < 0.01, *** *p* < 0.001, **** *p* < 0.0001).

**Fig 6:**
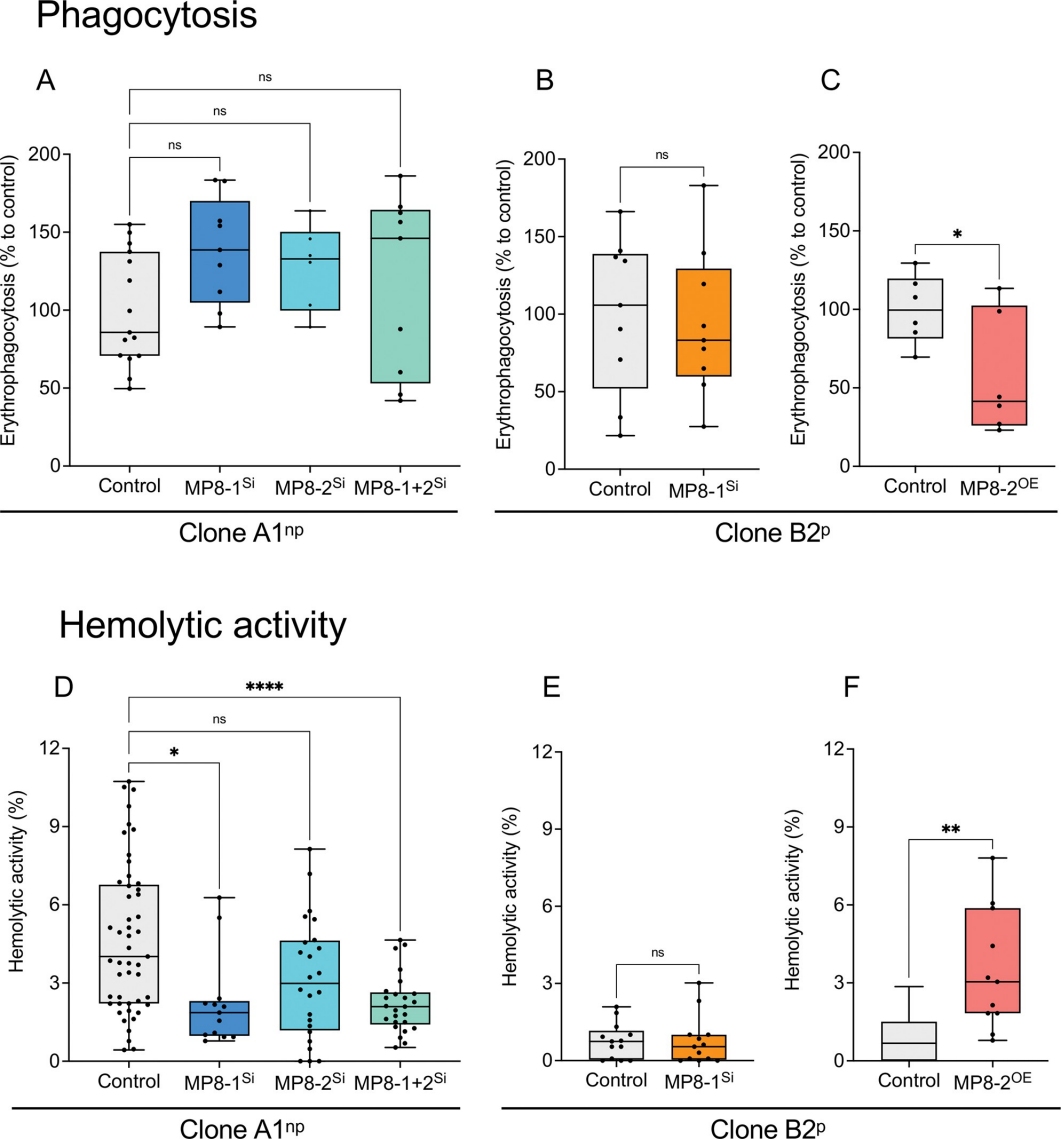
Determination of erythrophagocytosis (A-C) and hemolytic activity (D-F) of silencing transfectants A1^np^MP8-1^Si^, A1^np^MP8-2^Si^, A1^np^MP8-1+2^Si^ and B2^p^MP8-1^Si^ and overexpression transfectant B2^p^MP8-2^OE^. Non-transfected A1^np^ and B2^p^ trophozoites were used as controls for silencing transfectants and B2^p^ trophozoites transfected with the control plasmid pNC were used as control for overexpression transfectants. To determine erythrophagocytosis (A-C), trophozoites (2 x 10^5^) and erythrocytes (2 x 10^8^) were incubated for 30 min at 37°C, non-phagocytosed erythrocytes were lysed, then the amoebae were lysed in 1% Triton-X-100 and absorbance was measured at 405 nm. The mean value of the controls was defined as 100% and the measured OD_405 nm_ values of the samples were related to it. At least six biological replicates were performed. For hemolytic activity (D-F), 1.25 x 10^5^ trophozoites were mixed with 2.5 x 10^8^ erythrocytes in 1 ml of PBS and incubated at 37°C for 1 h. After incubation, the cells were sedimented, and the hemoglobin released in the supernatant was measured at 530 nm. Separately incubated erythrocytes and trophozoites were used as negative controls. To determine 100% hemoglobin release, 2.5 x 10^8^ erythrocytes were lysed in 1 ml of water. Experiments were performed at least 3 times in quadruplicate. Significance was determined using one-way ANOVA (A, D) and unpaired *t* test (B/C; E/F) (ns: not significant, *p < 0.05, **p < 0.01, **** *p* < 0.0001).

To obtain information on the effect of silencing and overexpression on the phenotype of *E*. *histolytica*, the motility, growth, phagocytosis rate, hemolytic activity, cytopathic activity, and cysteine peptidase activity of the transfectants were analyzed. Although silencing affects the expression of a number of genes, both silencing and overexpression have little effect on trophozoite motility, with significantly increased motility (*p*adj = 0.0347) observed for A1^np^EhMP8-2^Si^ transfectants (Fig 5A–5C and S2 Prism). To determine the growth rate of the different transfectants, the division rate was determined in 24 h periods (Fig 5D–5F and S3 Prism). In contrast to the motility, silencing of *ehmp8-1* resulted in significant inhibition of growth in both non-pathogenic A1^np^ and pathogenic B2^p^ trophozoites (*p*adj = 0.0008, *p* < 0.0001) (Fig 5D and 5E and S3 Prism). Similarly, overexpression of *ehmp8-2* in B2^p^ resulted in significantly faster growth (*p* = 0.0031) (Fig 5F and S3 Prism). Significantly decreased erythrophagocytosis (*p* = 0.0414) was observed only for B2^p^EhMP8-2^OE^ transfectants (Fig 6A–6C and S4 Prism). Hemolytic activity is significantly reduced in A1^np^EhMP8-1^Si^ (*p*adj = 0.0165) and A1^np^EhMP8-1+2 (*p*adj < 0.0001) (Fig 6D and S5 Prism), whereas no effect was observed in B2^p^EhMP8-1^Si^ transfectants (Fig 6E and S5 Prism). However, B2^p^ trophozoites already have very low hemolytic activity (Fig 6E and S5 Prism). Overexpression of the *ehmp8-2* gene in pathogenic B2^p^ trophozoites led to a significant increase in hemolytic activity (B2^p^MP8-2^OE^, *p* = 0.0024) (Fig 6F and S5 Prism).

**Fig 7:**
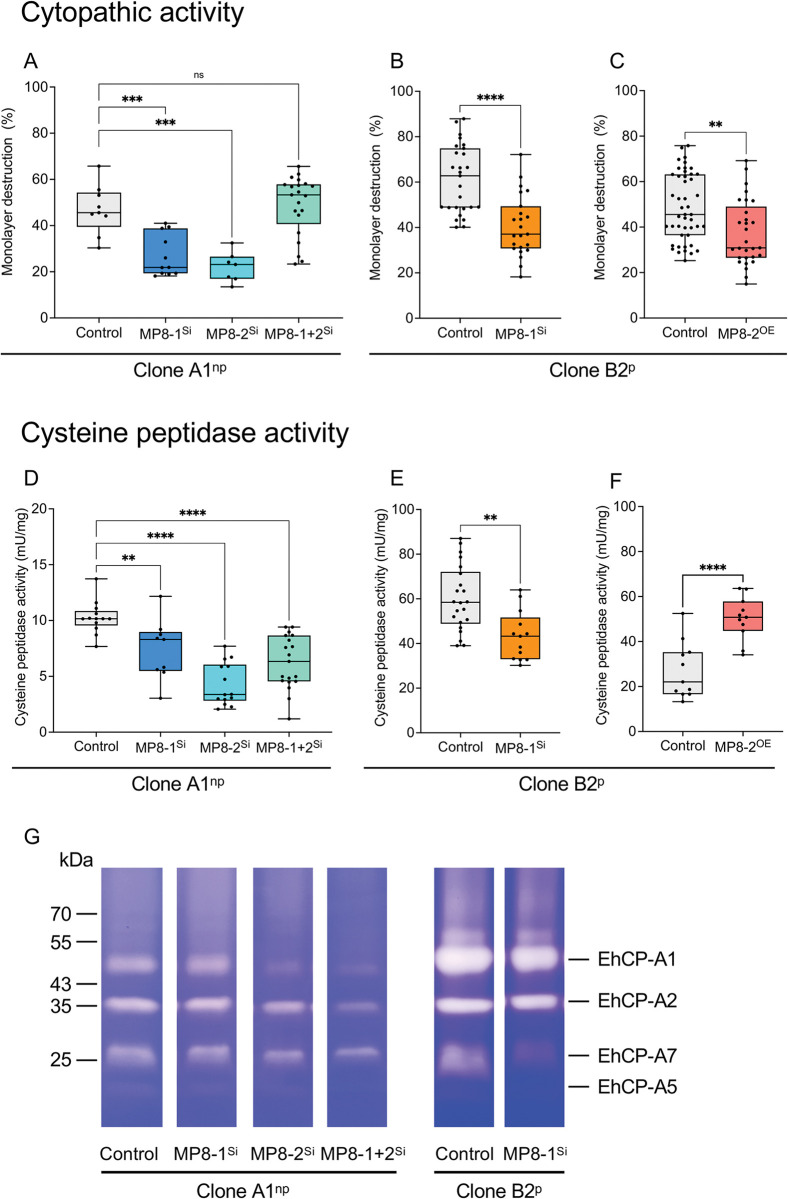
Determination of cytopathic activity (A-C) and cysteine peptidase activity (D-G) of silencing transfectants A1^np^MP8-1^Si^, A1^np^MP8-2^Si^, A1^np^MP8-1+2^Si^ and B2^p^MP8-1^Si^ and overexpression transfectant B2^p^MP8-2^OE^. Non-transfected A1^np^ and B2^p^ trophozoites were used as controls for silencing transfectants and B2^p^ trophozoites transfected with the control plasmid pNC were used as control for overexpression transfectants. To determine cytopathic activity (A-C), HepG2 cells (1 x 10^5^) were seeded in 24 well plates, cultured for 48 h and stained with BCECF. Subsequently, 1 x 10^5^ trophozoites were added to the cells in 500 μl DMEM medium and incubated at 37° for 1 h. Afterwards, the cells were lysed, centrifuged and the supernatant was measured at 485 nm absorbance and 535 emission. The negative control was set at 100%. Experiments were performed at least 3 times in triplicate. Cysteine peptidase activity (D-G) was determined using Z-Arg-Arg-pNA as substrate. Experiments were performed at least 6 times in duplicate. Significance was determined using one-way ANOVA (A, D) and unpaired *t* test (B/C; E/F) (ns: not significant, **p < 0.01, *** *p* < 0.001, **** *p* < 0.0001). (G) Determination of cysteine peptidase activity using substrate gel electrophoresis. 4 μg of amoeba extracts from controls (A1^np^/B2^p^) and transfectants (A1^np^MP8-1^Si^, A1^np^MP8-2^Si^, A1^np^MP8-1+2^Si^, B2^p^MP8-1^Si^) were separated on SDS-Page co-polymerized with gelatine. To visualize the cysteine peptidase activity, the gels were stained with Coomassie Blue.

Silencing of *ehmp8-1* or *ehmp8-2* alone in both clones A1^np^ and B2^p^ resulted in a significant reduction in cytopathic activity (cell monolayer destruction) between 33%– 53% (A1^np^EhMP8-1^Si^, *p*adj = 0.0007; A1^np^EhMP8-2^Si^, *p*adj = 0.0002; B2^p^EhMP8-1^Si^, *p =* 0.0014) (Fig 7A and 7B and S6 and S7 Prism). However, silencing of both metallopeptidase genes in A1^np^ trophozoites had no effect on cytopathic activity (Fig 7A and S6 Prism). Surprisingly, overexpression of *ehmp8-2* in B2^p^ trophozoites (B2^p^MP8-2^OE^) also led to a significant reduction of the monolayer destruction (*p* = 0.0014) (Fig 7C and S7 Prism). Furthermore, silencing led to a significant reduction in cysteine peptidase activity in all silencing transfectants examined (A1^np^EhMP8-1^Si^, *p*adj = 0.01; A1^np^EhMP8-2^Si^, *p*adj < 0.0001; A1^np^EhMP8-1+2^Si^, *p*adj < 0.0001; B2^p^EhMP8-1^Si^, *p* = 0.0012) (Fig 7D and 7E and S8 Prism). The cysteine peptidase activity in A1^np^ transfectants decreased from 10.3±1.4 (control) to 7.4±2.7 mU/mg (A1^np^EhMP8-1^Si^), 4.3±1.9 mU/mg (A1^np^EhMP8-2^Si^), and 6.2±2.4 mU/mg (A1^np^EhMP8-1+2^Si^) (Fig 7D and S8 Prism). The cysteine peptidase activity of B2^p^ trophozoites was 60±15 mU/mg and decreased to 43±11 mU/mg after silencing of *ehmp8-1* (Fig 7E and S8 Prism). In contrast, overexpression of *ehmp8-2* in B2^p^ trophozoites resulted in a significant increase in cysteine peptidase activity (B2^p^MP8-2^OE^, *p* < 0.0001) (Fig 7F and S8 Prism). Substrate gel electrophoresis confirmed the results of the activity measurement. The overall decrease in activity of the A1^np^MP8-1^Si^ transfectant compared to the control is too small to be detected in a substrate gel. However, for the A1^np^MP8-2^Si^ transfectant, though, there is a clear decrease in the intensity of the EhCP-A1 and EhCPA-7 bands, and for the A1^np^MP8-1+2^Si^ transfectant, all bands show a decrease in intensity. A similar effect was observed for the B2^p^MP-1^Si^ transfectants. In particular, the activity bands of EhCP-A7 show a reduced in intensity (Fig 7G). However, the regulation seems to occur at the translational level, as the expression of *ehcp-a1*, *ehcp-a2*, *ehcp-a5* and *ehcp-a7* was not significantly altered in the different transfectants compared to the controls. The only exception is *ehcp-a7*, which is significantly upregulated two-fold in the A1^np^MP8-1^Si^ transfectant compared to the control (*p*adj = 0.023) (S1, S2, S3, S7 Tables).

### EhMP8-1 and EhMP8-2 are localized in trophozoite vesicles

To determine the localization of the two metallopeptidases, non-pathogenic A1^np^ trophozoites were transiently transfected with plasmids allowing translation of the respective protein fused to a c-Myc tag at the C-terminus. Immunofluorescence assays with wild-type B2^p^ trophozoites used as controls show that the α-myc antibody did not detect any protein in the cytoplasm of saponin-treated cells or on the surface of trophozoites not treated with saponin (S2 Fig).

**Fig 8:**
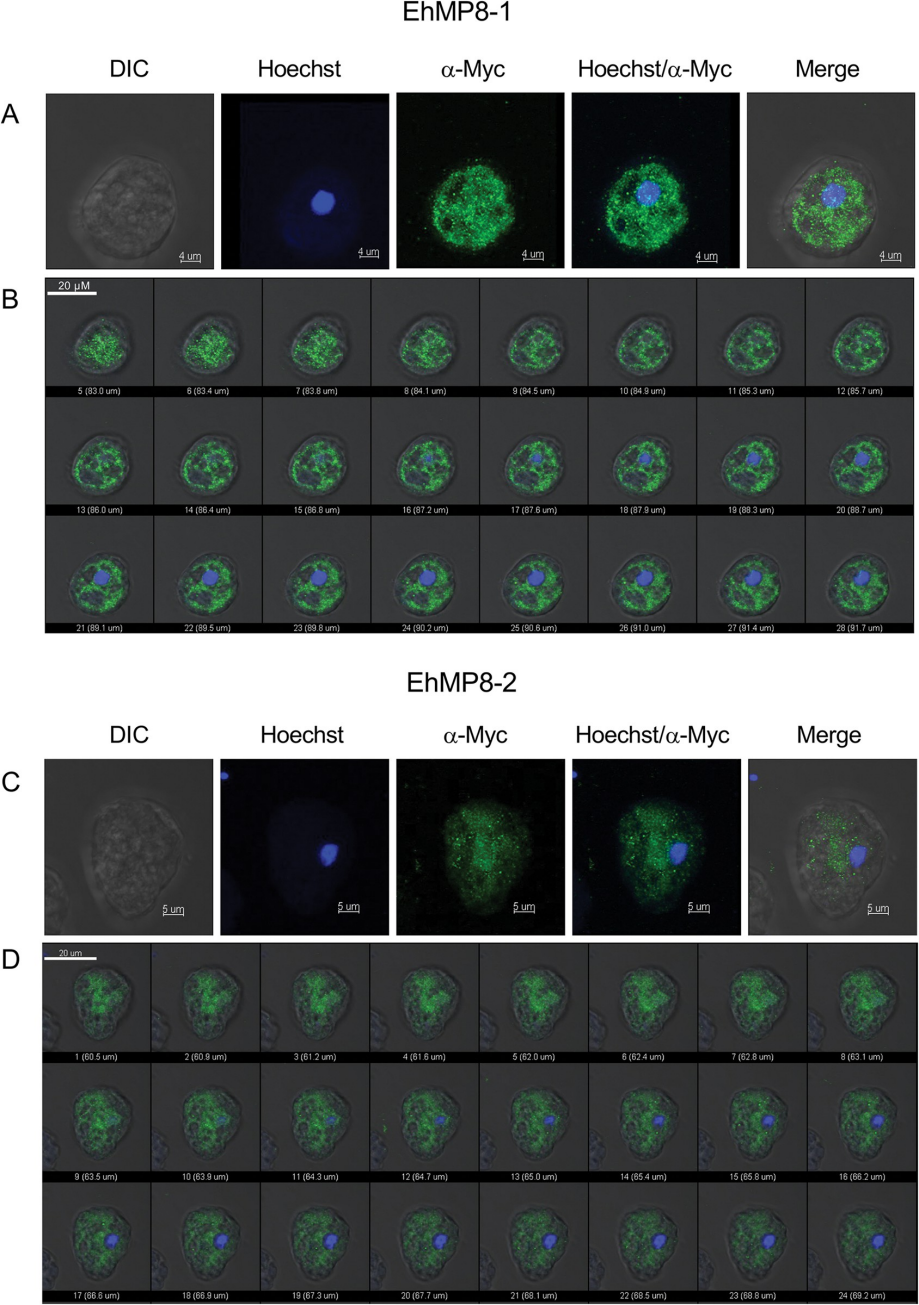
Microscopic analysis of A1^np^MP8-1^Myc^ and A1^np^MP8-2^Myc^ transfectants for localization of the metallopeptidases EhMP8-1 and EhMP8-2. The A1^np^ trophozoites were transfected with the expression plasmids pNCMP8-1^Myc^ or with pNCMP8-2^Myc^, which allowed the production of a metallopeptidase-myc fusion protein, and the myc-tag could be stained with a specific α-myc antibody. For the immunofluorescent analysis, trophozoites were fixed with PFA and permeabilized with saponin. MP8-1^Myc^ and MP8-2^Myc^ fusion proteins were stained with ⍺-c-myc primary antibody (1:100) and ⍺-mouse Alexa Fluor 488 (1:400, green). A, C. Single trophozoite of the A1^np^MP8-1^Myc^ (A) and A1^np^MP8-2^Myc^ transfectant (C). Shown are transmitted light images (DIC), staining of nuclei with Hoechst dye, staining of EhMP8-1 and EhMP8-2 with α-Myc antibody, and an overlay of the images (merge). B, D. Single images of confocal micrographs of a trophozoite of the A1^np^MP8-1^Myc^ (B) and A1^np^MP8-2^Myc^ (D) transfectant. The single images show 0.38 μm thick sections through the trophozoites. A total of 42 images were obtained, and only every second image is shown here.

In the A1^np^ transfectants A1^np^MP8-1^Myc^ and A1^np^MP8-2^Myc^, both metallopeptidases were detected in vesicle-like structures within the trophozoites. They were mainly localized in vesicles in the endoplasm of trophozoites (Fig 8A–8D). Neither EhMP8-1 nor EhMP8-2 were detected on the cell surface of trophozoites (S4A–S4C Fig).

### Comparison of EhMP8-1 and EhMP8-2 with leishmanolysins and invadolysins of different organisms

Comparing the amino acid sequences of EhMP8-1 and EhMP8-2 with leishmanolysin-like peptidases from different organisms revealed that the percent identity to those of the liver fluke *Clonorchis sinensis*, the avian protozoan parasite *Histomonas meleagridis*, and *T*. *vaginalis* ranges from 16% to 20%. The identity to leishmanolysin from *L*. *major* is 22% to 23%. The highest identities (24% to 28%) were found with various metazoan invadolysins such as those from the hookworm *Ancylostoma caninum*, the whipworm *Trichuris suis*, *Drosophila melanogaster*, *Mus musculus*, *Macaca thibetana*, humans, and the soil-dwelling amoeba *Dictyostelium discoideum* (S8 Table).

### Overexpression, but not silencing, of *ehhp127* affects the expression of other genes

**Fig 9:**
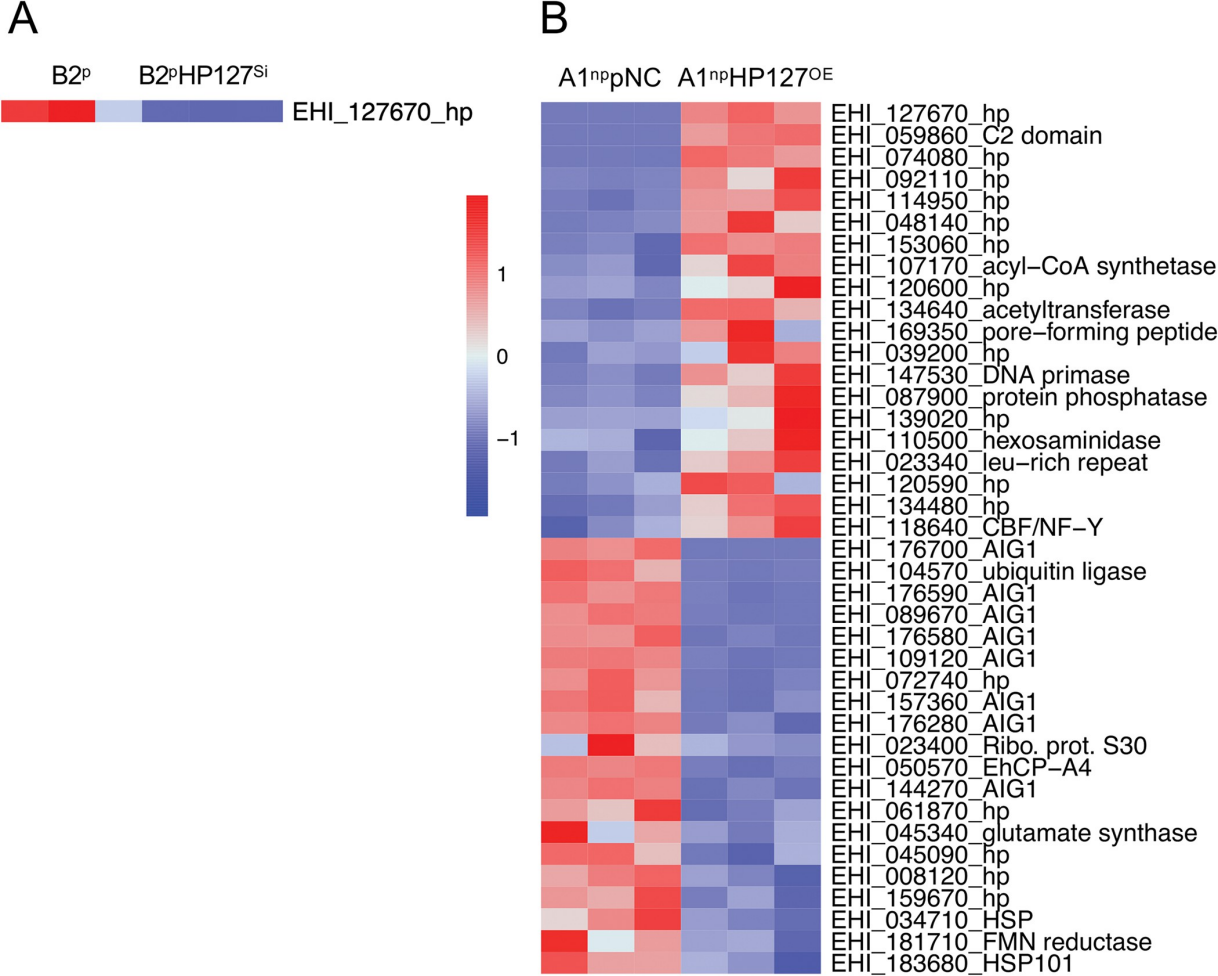
Heatmap of genes significantly differentially expressed (> 1.8 fold, *p*adj < 0.05) in the different *E*. *histolytica* transfectants silencing or overexpressing *ehhp127* compared to the corresponding controls. A maximum of 20 up- and downregulated genes with the highest fold change are shown. A. B2^p^ (control) versus B2^p^HP127^Si^, B. A1^np^pNC (control) versus A1^np^HP127^OE^.

It has been shown that *ehhp127* is almost exclusively expressed in clone B2^p^ (reads: B2^p^-2314.73 vs A1^np^ 12.01) [4]. In contrast to the silencing of *ehmp8-1* and *ehmp8-2* genes, the silencing of *ehhp127* in B2^p^ trophozoites (B2^p^HP127^Si^) had no effect on the expression of other genes (Figs 9A and S3 and S9 Table). However, this is different when *ehhp127* was overexpressed in clone A1^np^ (Figs 9B and S3 and S9 Table). In A1^np^HP127^OE^ transfectants, *ehhp127* was overexpressed approximately 5500-fold. Overall, 17 genes were significantly upregulated more than 3-fold and 40 genes were significantly upregulated more than 2-3-fold (*p*adj < 0.05) (Figs 9B and S3 and S10 Table). However, when the expression profile of the control transfectant A1^np^pNC is compared to that of non-transfected A1^np^ trophozoites, we observed 242- and 7-fold repression of the six most differentially expressed genes, whereas in A1^np^HP127^OE^ transfectants, the expression level returns to that of wild-type A1^np^ [4]. All six genes encode for hypothetical proteins (S10 Table). The reasons for this effect in mock transfection are unknown.

Only one of the additional differentially expressed genes (*ehi_062680*), encoding a hypothetical protein, was also found to be significantly differentially expressed between wild-type A1^np^ and B2^p^ trophozoites. This gene was 20-fold more highly expressed in B2^p^ than in A1^np^ trophozoites [4]. The increase in expression from A1^np^pNC to A1^np^HP127^OE^ was 2.5-fold (S10 Table). However, transfection with the control plasmid pNC also appears to affect expression. The expression of a number of genes was up- or down-regulated compared to wild-type A1^np^, but also compared to A1^np^Eh127^OE^ transfectants (S10 Table). In total, the expression of 86 genes was significantly downregulated. Of these, 43 genes are downregulated >3-fold and another 43 up to 2-3-fold (S10 Table). Again, an effect of the mock transfection can be seen. The expression of 18 genes that were normally not or only weakly expressed in A1^np^ wild-type trophozoites occurs in A1^np^pNC. However, the expression profile of the A1^np^HP127^OE^ transfectants has returned to the low levels seen in A1^np^ trophozoites. The difference in expression here was between 4200- and 6.7-fold. Except for one *aig1* gene and one gene encoding a putative DNA polymerase, these are all genes encoding hypothetical proteins (Table 1). For all other genes that were significantly downregulated in A1^np^HP127^OE^ transfectants, the expression in A1^np^pNC control transfectants correlated with that in A1^np^ wild-type trophozoites [4]. It is therefore likely that this is a specific effect of *ehhp127* overexpression. It is striking that, overall, the expression of 11 *aig1* genes was significantly downregulated (Table 1). Furthermore, the expression of the genes encoding cysteine peptidases *ehcp-a4* and *ehcp-a6*, genes encoding heat shock proteins (HSP101, DNAJ family protein, HSP70) and genes encoding antioxidants (peroxiredoxin, iron-sulfur flavoprotein) was downregulated in A1^np^HP127^OE^ transfectants (Figs 9B and S3 and S10 Table).

For nine genes, the expression profile of A1^np^pNC transfectants compared to A1^np^HP127^OE^ transfectants was comparable to that of A1^np^ to B2^p^ trophozoites. In both A1^np^HP127^OE^ transfectants and B2^p^ trophozoites, these genes are less expressed compared to A1^np^pNC control transfectants and A1^np^ trophozoites, respectively. These nine genes encode for heat shock proteins (EHI_034710, EHI_022620), AIG family proteins (EHI_126560, EHI_126550), DNA mismatch repair protein Msh2 (EHI_123830), splicing factor 3B subunit 1 (EHI_049170) and three hypothetical proteins (EHI_005657, EHI_075640, EHI_075690) (Figs 9B and S3 and S10 Table).

Overexpression of *ehhp127* significantly regulated only one GO-BP term, namely, "obsolete electron transport" (S11 Table). No significantly regulated pathway was identified within GO-CC. This is different for GO-MF, where 20 pathways are affected, including “guanyl ribonucleotide binding”, “lysozyme activity”, and “FMN reductase activity” (S11 Table).

### Altered expression of *ehhp127* has a major impact on motility and cytopathic activity

As for the EhMP8 transfectants, various phenotypic characteristics were also analyzed for the *ehhp127* overexpressing and silencing transfectants (A1^np^HP127^OE^, B2^p^HP127^Si^).

**Fig 10:**
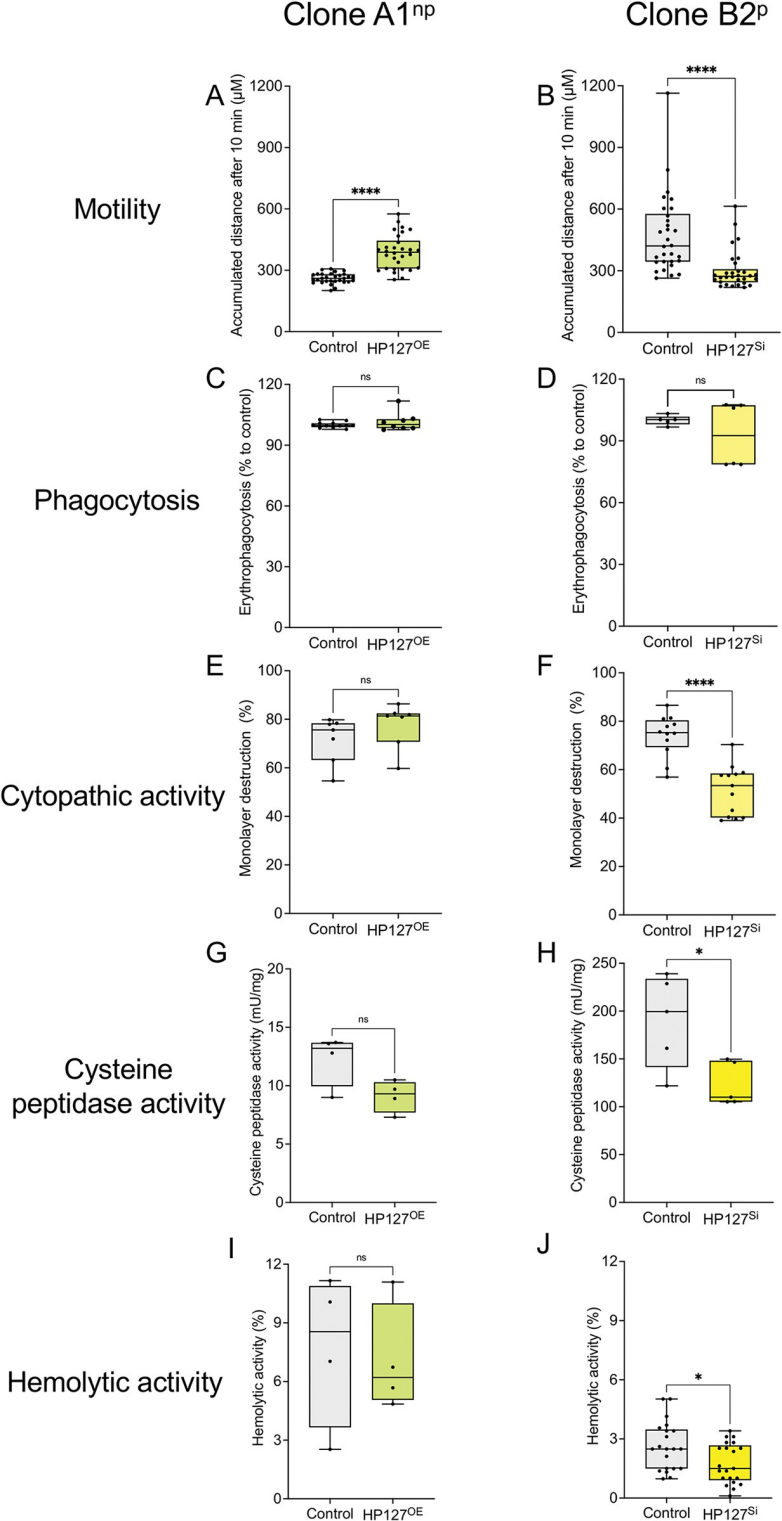
Determination of motility (A, B), erythrophagocytosis (C, D), cytopathic activity (E, F), cysteine peptidase activity (G, H), and hemolytic activity (I, J) of EhHP127 transfectants (overexpressing transfectant: A1^np^HP127^OE^, pNC-transfected A1^np^ trophozoites were used as control; silencing transfectant: B2^p^HP127^Si^, non-transfected B2^p^ trophozoites were used as control). A, B. To determine motility, the accumulated distance (μm) was measured after 10 min. For each transfectant/control 30 amoebae (two biological replicates, 15 amoebae each) were analyzed. Significance was determined using the unpaired *t* test (****p < 0.0001). C, D. To determine erythrophagocytosis, trophozoites (2 x 10^5^) and erythrocytes (2 x 10^8^) were incubated for 30 min at 37°C, non-phagocytosed erythrocytes were lysed, then the amoebae were lysed in 1% Triton-X-100 and absorbance was measured at 405 nm. The mean value of the controls was defined as 100%, and the measured OD_405 nm_ values of the samples were related to 100%. At least five biological replicates were performed per transfectant/control and significance was determined using the unpaired *t* test (ns: not significant). E, F. To determine cytopathic activity, HepG2 cells (1 x 10^5^) were seeded in 24 well plates, cultured for 48 h and stained with BCECF. Subsequently, 1x10^5^ trophozoites were added to the cells in 500 μl DMEM medium and incubated for 1 h at 37°C. Afterwards, the cells were lysed, centrifuged and the supernatant was measured at 485 nm absorbance and 535 nm emission. The negative control was set at 100%. Experiments were performed at least 3 times in duplicate. Significance was determined using the unpaired *t* test (ns: not significant, ****p < 0.0001). G, H. Cysteine peptidase activity was determined using Z-Arg-Arg-pNA as substrate. The experiments were performed three times in duplicate for A1^np^HP127^OE^ (G) and five times in duplicate for B2^p^HP127^Si^ (H). Significance was determined using the unpaired *t* test (ns: not significant, *p < 0.05). I, J. To determine hemolytic activity, 1.25 x 10^5^ trophozoites were mixed with 2.5 x 10^8^ erythrocytes in 1 ml PBS and incubated at 37°C for 1 h. After incubation, the cells were sedimented and the hemoglobin released in the supernatant was measured at 530 nm. Separately incubated erythrocytes and trophozoites were used as negative controls. To determine 100% hemoglobin release, 2.5 x 10^8^ erythrocytes were lysed in 1 ml of water. The experiment was performed 2 times in duplicate (I) and 7 times in triplicate (J). Significance was determined using the unpaired *t* test (ns: not significant, *p < 0.05).

Silencing and overexpression of *ehhp127* had less overall impact and less significant effects on the *E*. *histolytica* phenotype. For the silencing transfectants, this was not surprising since no other genes except *ehhp127* itself were affected in its expression. What is surprising, however, is that although the expression profile for a number of genes was regulated during overexpression, this only affected motility (Fig 10A and S9 Prism). This was also highly significantly affected in silencing transfectants (Fig 10B and S9 Prism). While overexpression of *ehhp127* in A1^np^ (A1^np^HP127^OE^) increases the distance traveled from 261±25 μm to 389±84 μm after 10 min (*p* < 0.0001) (Fig 10A and S9 Prism), silencing of *ehhp127* in B2^p^ (B2^p^HP127^Si^) led to a decrease in the distance traveled from 449±141 μm/10 min to 270±38μm/10 min (*p* < 0.0001) (Fig 10B and S9 Prism). Erythrophagocytosis was not affected in the *ehhp127* transfectants A1^np^HP127^OE^ and B2^p^HP127^Si^ (Fig 10C and 10D and S10 Prism). However, as with all metallopeptidase transfectants, we observed that silencing of *ehhp127* reduced cytopathic activity. While 74±8.6% of the monolayer was destroyed by the control (B2^p^ trophozoites), silencing of the *ehhp127* gene (B2^p^HP128^Si^) reduces the destruction to 51±10% (*p* < 0.0001) (Fig 10F and S11 Prism). As previously described [5], overexpression had no significant effect on cysteine peptidase activity (Fig 10G and S12 Prism), whereas silencing of *ehhp127* results in a significant reduction of cysteine peptidase activity (190±49 mU/mg vs 123±23 mU/mg, *p* = 0.024) (Fig 10H and S12 Prism). With respect to hemolytic activity, only a significant reduction in B2^p^ (2.6±1.2% vs 1.7±1%, *p* = 0.015) was observed after *ehhp127* silencing (Fig 10J and S13 Prism).

### Localization of EhHP127 in vesicles of trophozoites

**Fig 11:**
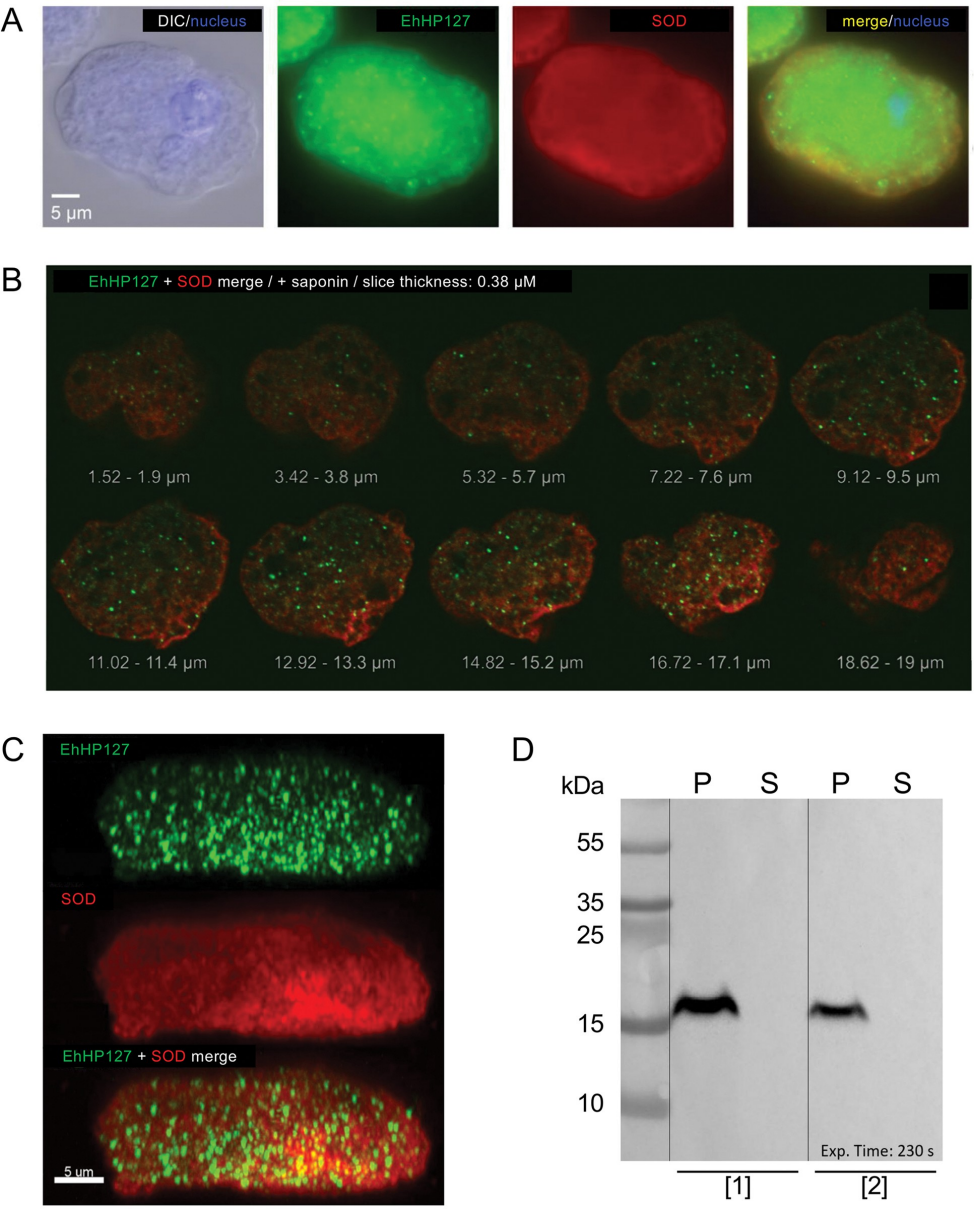
Localization of the hypothetical protein EhHP127. B2^p^ trophozoites were transfected with the expression plasmid pNCHP127^Myc^, which allowed the production of an EhHP127-Myc fusion protein, and the myc-tag was stained with a specific α-myc antibody. A-C. For the immunofluorescent analysis, trophozoites were fixed with PFA and permeabilized with saponin. EhHP127^Myc^ fusion protein was stained with ⍺-c-myc primary antibody (1:100) and α-mouse Alexa Fluor 488 (1:400, green). For co-localization an antibody against the cytoplasmically localized superoxide dismutase (SOD) and α-rabbit Alexa Fluor 594 (1:400, red) was used. Nuclei were stained with Hoechst dye (blue). A. Single trophozoite from the B2^p^HP127^Myc^ transfectant. B. Confocal images of a trophozoite of the B2^p^Eh127Myc transfectant. Series of 10 sections with a distance of 1.9 μm from top to bottom, covering a major part of the cell. C. Side view of the whole cell with the three modes green, red and merge, for the detection of EHI_127670 and SOD both single and merged. For the side view, a 3D-model was constructed out of 55 single slices using Imaris. D. Western blot of a 13% Tricine-SDS-PAGE using an ⍺-c-myc primary antibody (1:1000) and ⍺-mouse-HRP secondary antibody (1:2000). For the Western blot, B2^p^ trophozoites were transfected twice independently ([1], [2]) and the pellet fraction (P) and the soluble fraction (S) were prepared from each of the two transfectants.

Similar to the experiments in which the hypothetical protein EhHP127 was overexpressed, amoebae were transiently transfected with a plasmid allowing translation of EhHP127 fused to a c-Myc tag at the C-terminus. In B2^p^EhHP127^Myc^ transfectants, EhHP127^Myc^ was also localized to trophozoite vesicles. A uniform distribution within the trophozoites was observed (Fig 11A–11C). In western blot analysis, EhHP127 was detected only in the insoluble pellet fraction and not in the soluble fraction (Fig 11D). However, EhHP127 was not detected on the surface of trophozoites (S4D Fig).

## Discussion

Comparative transcriptome analysis of pathogenic and non-pathogenic clones, all derived from the *E*. *histolytica* isolate HM-1:IMSS, identified, among others, two differentially expressed genes whose silencing or overexpression affects the virulence phenotype of the amoebae. Overexpression of the gene *ehmp8-2*, encoding the metallopeptidase EhMP8-2, was shown to decrease the virulence of the pathogenic clone B2^p^, whereas overexpression of the gene *ehhp127*, encoding a hypothetical protein, increased the virulence of the non-pathogenic clone A1^np^ in mouse infection experiments [4, 5]. To better understand the function of these two proteins, corresponding silencing or overexpression transfectants were generated and their phenotypes analyzed.

The two *E*. *histolytica* metallopeptidases, EhMP8-1 and EhMP8-2, belong to the M8 peptidase family (leishmanolysin-/gp63-like). The name is derived from leishmanolysin, an important pathogenicity factor of *Leishmania* [11]. This leishmanial peptidase is the most abundant protein on the cell surface during the promastigote stage of the parasite and acts as a ligand with various molecular partners [12–15]. Involvement in important processes of pathogenesis seems to be characteristic for different members of this M8 family of metalloproteases in various parasites [16–21]. However, leishmanolysin-like peptidases are not unique to parasitic protozoa, but are conserved in many different organisms, and the number of gene copies can vary substantially. Compared to the role of M8 family members in other protozoan parasites, it is surprising that non-pathogenic A1^np^ trophozoites express both genes, while pathogenic B2^p^ trophozoites still have two intact genes but no longer express the *ehmp8-2* gene [4]. Leishmanolysin-like proteins have also been described in metazoans such as *Drosophila melanogaster* or humans, where they are called invadolysins with only one representative each [22, 23]. The identity of EhMP8-1 and EhMP8-2 with leishmanolysins and invadolysins of other organisms is relatively low, namely 16%-28%. Interestingly, the metallopeptidases of *E*. *histolytica* show the highest identity to several invadolysins of metazoans (S8 Table). Invadolysins of *Drosophila* are involved in the development and show a complex cellular localization [22], namely in the nucleus and in structures surrounding lipid droplets [23].

We detected both EhMP8-1 and EhMP8-2 in vesicles in the amoebae (Fig 8A–8F). In contrast to Teixera and colleagues [7], who sporadically detected EhMP8-1 on the cell surface of amoebae, we could not verify this localization. It is not yet clear what kind of vesicles the *E*. *histolytica* metalloproteinases are found in. It is also not known whether they are lipid droplets. In resting macrophages, invadolysin was localised in lipid droplets in the cytoplasm, but in actively migrating macrophages it was highly concentrated at the leading edge of the cell, suggesting that invadolysins are involved in cell migration [22]. It has also been shown that inhibition of intracellular membrane trafficking blocks extracellular matrix degradation by invadopodia [24], and since endocytosis is associated with cell migration [25, 26], it is possible that lipid droplet-associated invadolysin affects cell migration via the invadopodia [23]. This parallels studies on EhMP8-1. Hasan and colleagues showed that silencing of *ehmp8-1* (*ehmsp-1*) renders trophozoites hyper-adherent and less motile. They also showed that silencing of *ehmp8-1* results in parasites that are unable to form specialized, dot-polymerized actin structures (F-actin) upon interaction with human fibronectin. These short-lived F-actin structures resemble those of mammalian cell invadopodia [27]. In contrast to the study by Hasan and colleagues, we did not find any effects of silencing *ehmp8-1* on motility; however, silencing *ehmp8-2* resulted in significantly reduced motility (Fig 5A). Furthermore, we showed that both silencing and overexpression of *ehmp8-2* and its homologous gene *ehmp8-1* have the most impressive effects on cysteine peptidase activity (Fig 7D–7F). Silencing leads to a decrease in the cysteine peptidase activity, whereas overexpression leads to an increase. The ability to destroy a monolayer is also significantly impaired, although both overexpression and silencing lead to a reduction in cytopathic activity (Fig 7A–7C).

To determine whether the altered phenotypes were indeed due to silencing or overexpression of the corresponding *ehmp8* genes, the expression profiles of the different transfectants were analyzed by RNAseq. It was striking that silencing *ehmp8-2*, but also of the homologous gene, *ehmp8-1*, or of both genes affected the expression of several hundred genes in the transfectants. It is not known why silencing has such a strong effect on the expression of many other genes. As many of these genes encode proteins of unknown function, it is impossible to determine the full impact on biological processes. Remarkably, the silencing of *ehmp8-1* or *ehmp8-2* led to the upregulation of genes encoding antioxidants, including genes encoding iron-sulfur flavoproteins. This is consistent with GO analysis, with the GO-BP term "obsolete electron transport" and the GO-MF terms "oxidoreductase activity" or "FMN reductase activity" being most strongly affected (Fig 4A and 4B and S1 and S2 Table). The observation that silencing of the two metallopeptidase genes regulates fewer of the phenotypic properties analyzed can be explained by the fact that fewer genes are affected in their expression. Although we cannot definitively say why silencing both metallopeptidase genes regulates fewer genes than the two single silencing approaches, this result suggests a complex interplay between the two metalloproteases. However, this surprising result of comparing the different effects of silencing individual metalloproteinase genes with those of both metalloproteinase genes makes it possible to take a closer look at the similarities. The main similarity is the reduction of cysteine peptidase activity in all silencing transfectants. The increase in cysteine peptidase activity in the overexpression transfectants further supports the idea of a direct dependency of cysteine peptidase activity on the metalloproteinase activity. This modification of cysteine peptidase activity probably occurs post-translationally. Thus, the metalloproteases could be the molecules that activate cysteine proteases in *E*. *histolytica* from the respective propeptidases by proteolytic cleavage. Comparable mechanisms by which other proteases control the activation of proteases have already been described in other systems (e.g. activaton of pancreas proteases, for review [28], activation of caspases, for review [29]), which makes our assumption likely).

In addition, the expression of numerous *aig1* genes is reduced in the different EhMP8 silencing transfectants (Table 1). An influence on the expression of *aig* genes is also seen in the EhHP127^OE^ overexpression transfectants. In these, the expression of ten *aig1* genes is significantly downregulated (Table 1). AIG1 proteins were first described in the context of plant immune defense [30]. The functional significance of the large family of *E*. *histolytica* AIG1 proteins, which are encoded by more than 45 genes, is not yet fully understood, although numerous studies have tackled this problem [2]. The *E*. *histolytica* AIG1 proteins show structural similarities to the GTPases of the immunity-associated protein (GIMAPS)/immune-associated nucleotide-binding protein (IAN) family of AIG1-like GTPases, which are conserved between vertebrates and angiosperm plants [31]. Comparison of a pathogenic with a non-pathogenic HM-1:IMSS cell line, from which the clones studied here were derived, showed that of 34 *aig1* genes detected, 18 genes were more highly expressed in the pathogenic cell line B, and only one gene was expressed at a higher level in the non-pathogenic cell line A [2]. Some of the AIG1 proteins were found to have specific functions. For example, it was shown that overexpression of the *aig1* gene EHI_176590 in strain HM-1:IMSS cl6 resulted in increased formation of cell surface protrusions and increased adhesion of trophozoites to human erythrocytes [32]. Overexpression of the *aig1* gene EHI_180390 in trophozoites of the non-pathogenic strain UG10 resulted in increased virulence [33]. Furthermore, co-culture of pathogenic HM-1:IMSS with *Escherichia coli* reduced virulence *in vitro* and downregulated *aig1* gene expression. In contrast, the virulence of strain UG10 co-cultured with *E*. *coli* was increased and *aig1* expression was upregulated [33]. It has also been shown that an *aig1* gene is upregulated in its expression in the presence of H_2_O_2_ [34]. In amoebae resistant to 12 μM metronidazole, ten *aig1* genes were differentially expressed [35]. Similarly, differential expression was detected during *E*. *histolytica* invasion of the mouse intestine [36], and *aig1* genes were downregulated in trophozoites isolated from an amoebic liver abscess [37]. Three members of the *aig* family have also been identified as cyst-specific genes [38]. Taken together, these individual reports suggest that regulation of *aig1* expression has a significant impact on pathogenicity and pathogenicity-associated phenotypes.

**Fig 12:**
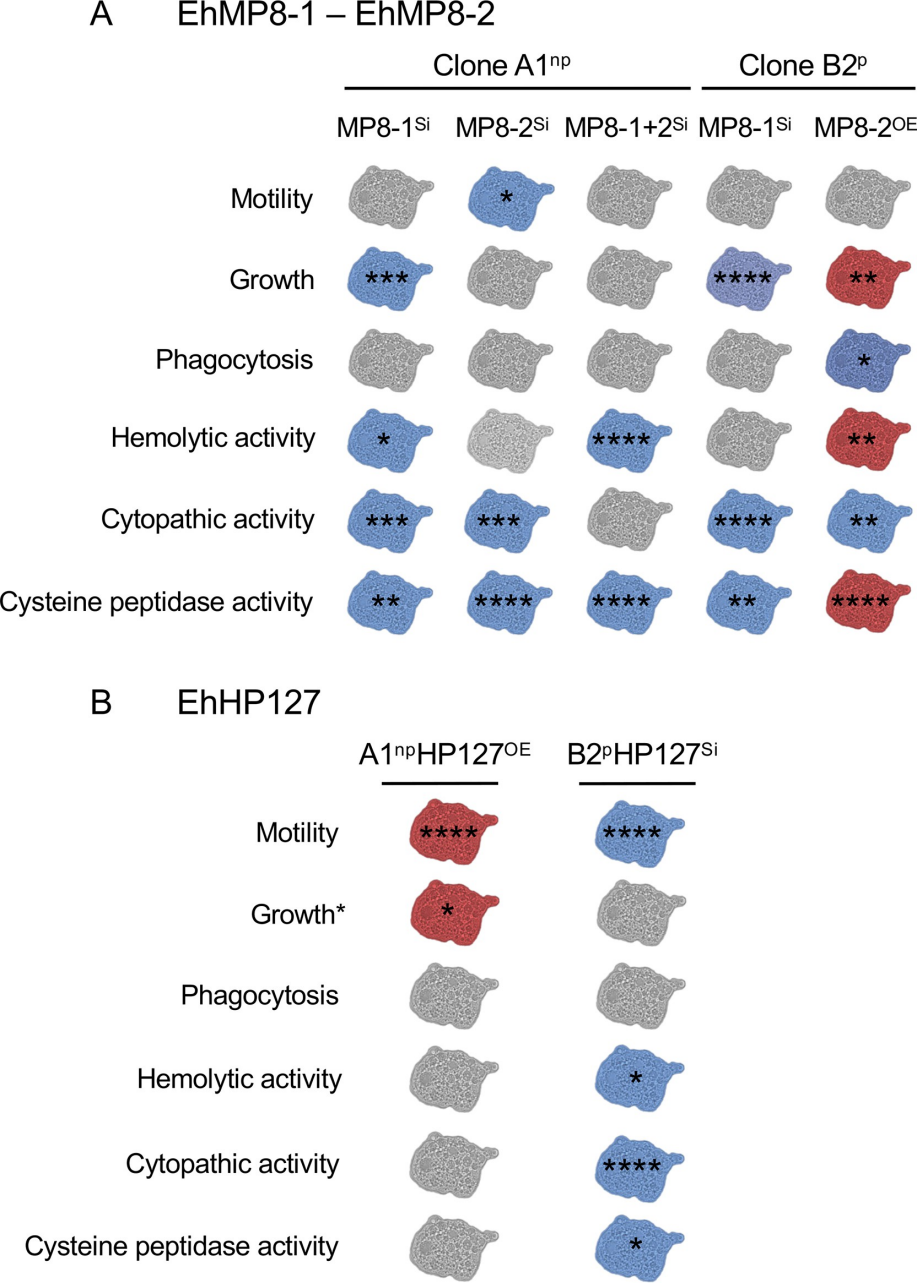
Schematic representation of phenotypic changes triggered by overexpression and silencing of EhMP8-1 and/or EhMP8-2 (A) and EhHP127 (B). Blue amoebae represent a decrease and red amoebae an increase in the phenotype under investigation. If there is no difference between controls and transfectants, the amoebae are shown in gray. In addition, the significance determined by a one-way ANOVA or an unpaired *t* test is shown (*p < 0.05, **p < 0.01, ***p < 0.001, ****p < 0.0001). *Growth, data from [5].

However, particularly in the EhHP127^OE^ overexpression transfectants, it is apparent that although the expression of ten *aig1* genes is downregulated, and the expression of a variety of other genes is altered, this has no significant effect on most of the phenotypes examined here. Overexpression of *ehhp127* only results in a significant increase in motility. In contrast, silencing of the *ehhp127*, which does not alter the expression of other genes, significantly reduces motility. It is therefore very likely that EhHP127 directly affects the motility of amoebae (Fig 12). Moreover, silencing also leads to a highly significant reduction in cytopathic activity. The destruction of a cell monolayer by *E*. *histolytica* is often attributed to its cysteine peptidase. However, this has been clearly refuted by several studies [39, 40]. It is more likely that the disruption of a cell monolayer is a mechanical effect due to the movement of the trophozoites, which might be the direct link to the cytopathic effect observed with the *ehhp127* transgenes. Silencing of *ehhp127* also results in reduced cysteine peptidase and hemolytic activity. That silencing of *ehhp127* has an effect on cysteine peptidase activity was previously shown by Matthiesen and colleagues and was confirmed here [5]. However, there is no information about the molecular and biological function of EhHP127. The gene has no clear homolog in the animal kingdom and no specific domains have been identified, making it impossible to hypothesize about its function.

The cytoplasm of *E*. *histolytica* contains a large number of vacuoles and vesicles. Very little is known about the proteome of these structures. In addition to the proteins studied here, EhHP127, EhMP8-1 and EhMP8-2, several cysteine peptidases and cysteine peptidase inhibitors have been detected in vesicle-like structures [41–47].

In summary, this study has provided some unexpected insights. First, the two genes *ehhp127* and *ehmp8-2*, which are differentially expressed between pathogenic and non-pathogenic clones, strongly influence the phenotypes associated with pathogenicity but appear to do so in a completely independent manner. The unknown gene *ehhp127* appears to primarily influence trophozoite mobility, whereas the metalloprotease *ehmp8-2* generates a highly complex and diverse response (Fig 12). The results of this study imply that both genes could act at different levels. While *ehhp127* influences the complex property of mobility and thus indirectly also pathogenicity, the metalloproteinases appear to influence cysteine protease activity directly and thus directly influence pathogenicity.

## Material and methods

### *E*. *histolytica* cell culture and generation of transfectants

Cultivation of *E*. *histolytica* trophozoites was performed under microaerophilic and axenic conditions at 37°C in plastic culture flasks (Corning, Kaiserslautern, Germany) using TYI-S-33 medium [48]. Non-pathogenic *E*. *histolytica* clone A1^np^ and pathogenic *E*. *histolytica* clone B2^p^ were derived from the cell lines HM-1:IMSS-A and HM-1:IMSS-B [4].

Overexpressing and silencing transfectants were generated as described [4, 5]. In summary, for overexpression of *ehmp8-2* (*ehi_042870*) in clone B2^p^ and *ehhp127* (*ehi_127670*) in clone A1^np^, trophozoites were transfected with the expression plasmid pNC containing the gene of interest under control of the *E*. *histolytica* lectin promoter (B2^p^MP8-2^OE^, A1^np^HP127^OE^). As a control B2^p^ or A1^np^ trophozoites were transfected with the plasmid pNC [4]. Overexpressing transfectants were cultured in TY-I-S-33 medium containing 20 μg/ml G-418 (Gibco, Thermo Fisher Scientific, Schwerte, Germany).

For silencing of *ehmp8-1* (*ehi_200230*) or *ehmp8-2* in clone A1^np^, trophozoites were transfected with the silencing plasmid pSiA containing *ehmp8-1* (267 bp) or *ehmp8-2* (1989 bp), respectively, in frame with the trigger region of *ehi_169670* (A1^np^MP8-1^Si^, A1^np^MP8-2^Si^). For silencing of *ehmp8-1* or *ehhp127* in clone B2^p^, trophozoites were transfected with the silencing plasmid pSiB containing *ehmp8-1* (268 bp) or *ehhp127* (917 bp) in frame with the trigger region of *ehi_074080* (B2^p^MP8-1^Si^) [5]. The silencing transfectants were grown in TY-I-S-33 medium containing 20 μg/ml G-418 for 3 weeks. After cloning of the transfectants by limited dilution, the cells were cultured without selection for at least 4 months until complete loss of the plasmid. Plasmid loss was proven by culturing the amoebae in the presence of 20 μg/ml G418 [5]. To produce the A1^np^EhMP8-1+2^Si^ transfectant, after successful silencing of *ehmp8-1*, the A1^np^EhMP8-1^Si^ transfectant was transfected with the silencing plasmid pSiA containing *ehmp8-2*. Subsequently, selection and cultivation were performed as described above. A1^np^ and B2^p^ trophozoites were used as controls. Overexpression and silencing were confirmed by specific qRT-PCR (S12 Table).

For localization studies, *ehhp127* (*ehi_127670*) was expressed under its own promoter using a myc-tag containing expression vector (pNC^Myc^) in clone B2^p^. The pNC^Myc^ plasmid is based on the pNC expression plasmid. A myc-tag was integrated into the *Bam*HI restriction site via *Bam*HI/*Bgl*II. To generate the pNC^Myc^ vector, two complementary oligonucleotides encoding the c-Myc-tag (Glu-Gln-Lys-Leu-Ile-Ser-Glu-Glu-Asp-Leu) and the restriction sites *Kpn*I, *Nhe*I, *Bam*HI, *Xho*I, and *Bgl*II at the 5’-end were hybridized (S12 Table). The resulting DNA fragment was then cloned into the pNC vector via the *Kpn*I restriction site at the 5’ end and the *Bgl*II restriction site at the 3’ end.

To generate the plasmid pNC_HP127^Myc^, a 500 bp long sequence upstream of the start ATG, presumably containing the promoter region, and the entire open reading frame of *ehi_127670* was amplified by PCR using a forward primer containing a *Kpn*I restriction site and the reverse primer containing an *Bam*HI restriction site (S12 Table). DNA from the clone B2^p^ was used as a template. The amplified insert was cloned into the pNC^Myc^ vector with *Kpn*I and *Bam*HI restriction sites.

Expression of *ehmp8-1* and *ehmp8-2* was also performed under their own promoter (pNCMP8-1^Myc^, pNCMP8-2^Myc^). For this purpose, a 500 bp long sequence upstream of start ATG, which presumably contains the promoter region, and the entire open reading frame of *ehmp8-1* (*ehi_200230)* and (*ehmp8-2) ehi_042870* was amplified by PCR using the forward primer containing a *Kpn*I restriction site and the reverse primer containing a *Bam*HI restriction site (S12 Table).

### RNA extraction and quantitative real-time PCR (qRT-PCR)

For RNA isolation, trophozoites were harvested, washed twice with sodium phosphate-buffered saline (NaPBS; 4°C; 6.7 mM NaHPO_4_, 3.3 mM NaH_2_PO_4_, 140 mM NaCl, pH 7.2) and lysed with Trizol reagent (QIAzol Lysis reagent, QIAgen, Hilden, Germany). Total RNA isolation and DNA digestion were performed using the Direct-zol RNA MiniPrep kit (Zymo Research, Irvine, CA, USA). All steps were performed according to the manufacturer’s instructions. RNA concentration and purity were determined using the NanoDrop 2000 (Thermo Fisher Scientific, Schwerte, Germany). The cDNA required for qRT-PCR was synthesized from the isolated RNA using the SuperScriptIII First-Strand Synthesis System Kit (Invitrogen, Thermo Fisher Scientific, Dreieich, Germany), according to the manufacturer’s instructions.

Sense and antisense primers were designed for qRT-PCR experiments to amplify 80–120 bp fragments of the genes of interest (S12 Table). The Luna Universal qPCR Master Mix kit (New England Biolabs, Frankfurt, Germany) was used to perform qRT-PCR. 2–4 biological replicates were analyzed in duplicate each time. Relative concentrations in gene expression were calculated using the 2^-ΔΔCT^ method and Rotor Gene software (Rotor Gene 6, Corbett Research). A1^np^, B2^p^, and B2^p^pNC were used as calibrators (control), and were set to 1. Actin was used as a housekeeping gene for normalization.

### Next generation sequencing

The quality of purified RNA was assessed using the Agilent 2100 Bioanalyzer System (Agilent Technologies, Santa Clara, CA, United States) and the Agilent RNA 6000 Pico Reagents Kit (Agilent Technologies). Ribosomal RNA was removed using the QIAseq FastSelect RNA Removal Kit (Qiagen, Hilden, Germany) according to the manufacturer’s instructions. RNA from each sample was prepared for sequencing using the QIAseq Stranded mRNA Library Kit (Qiagen, Hilden, Germany) according to the manufacturer’s instructions. Normalized libraries were pooled and sequenced using a NextSeq 500/550 Mid OutputKit v2.5 (Illumina, San Diego, CA, USA) with 150 cycles (2 × 75 bp paired-end) on a NextSeq 550 platform, generating a depth of 5–16 million paired-end reads for each sample. Reads were trimmed and filtered using Trimmomatic [49], and reads were aligned to the *E*. *histolytica* transcriptome (AmoebaDB, Release 61, 15 Dec 2022) using RSEM [50] and Bowtie2 [51] software. Differential expression was tested using DEseq2 to normalize the raw data [52].

### Determination of motility

24 h before the experiment, 2.5x10^5^ trophozoites were seeded into a T25 culture flask (Sarstedt, Nümbrecht, Germany). For the metallopeptidase transfectants three biological replicates were used and the speed of movement was determined for 20 amoebae each using an Evos FL Auto microscope (Thermo Fisher Scientific). For the Eh127 transfectants two biological replicates were used and the movement was determined for 15 amoebae each. A video was recorded for 10 min (one frame every 5 sec). Manual tracking of the amoebae was performed using ImageJ version 2.0.0-rc-43/1.51d with plugins for manual tracking and chemotaxis.

### Erythrophagocytosis assay

Human erythrocytes (blood group 0+ donated from the blood bank of the University Medical Center Hamburg-Eppendorf) and trophozoites to be examined were washed twice with incomplete TY-I-S-33 medium (200 x *g*, 10 min, 4°C). Subsequently, 2 x 10^8^ erythrocytes and 2 x 10^5^ trophozoites (ratio 1000:1) were added to a 5 ml tube with a total volume of 400 μl incomplete TY-I-S-33 medium. After incubation at 37°C for 30 min, 1 ml each of ddH_2_O was added twice and incubated for 1 min to lyse the non-phagocytized erythrocytes. The cells were then centrifuged at 400 x *g* for 4 min in a 15 ml tube and washed twice with NaPBS. The amoebae were then lysed to release the hemoglobin of the phagocytosed erythrocytes. For this purpose, 1 ml of 1% Triton-X 100 was added to the trophozoites. For photometric measurement, 200 μl of the lysed trophozoites were pipetted into a 96-well plate and measured at 405 nm. The mean value of each control was defined as 100% and the measured OD values were referenced to this value. For the metallopeptidase transfectants at least six biological replicates were performed. For the Eh127 transfectants at least five biological replicates were performed.

### Determination of cytopathic activity (monolayer destruction)

To determine the cytopathic activity of *E*. *histolytica*, 1 x 10^5^ cells of the hepatocyte cell line HepG2 (Merck, Darmstadt, Germany) were seeded in the wells of a 24-well plate 48 h before the experiment and incubated at 37°C in 2 ml HepG2 cell culture medium (Advanced DMEM; Gibco, Thermo Fisher Scientific, Schwerte, Germany) supplemented with 10% fetal calf serum (FCS; Capricorn, Hamburg, Germany) and 1x penicillin/streptomycin (Capricorn), with the medium changed once after 24 h. After 48 h, a confluent cell monolayer was formed. Trophozoites were seeded into a T12.5 culture flask 24 h prior to the experiment so that a confluent monolayer was formed the next day. At the beginning of the experiment, HepG2 cell culture medium was removed from the HepG2 cells, which were then washed with NaPBS. To stain the HepG2 cells, 200 μL NaPBS + 10 μM of the fluorescent dye BCECF, AM (2’,7’-bis-(2-carboxyethyl)-5-(and-6)-carboxyfluorescein, acetoxymethyl ester) was added. The cells were incubated at 37°C for 30 min. Afterwards, BCECF/NaPBS was removed, and the cells were washed twice with NaPBS (37°C). Then, 1 × 10^5^ trophozoites in 500 μl were added to the stained HepG2 cells. As a negative control, the stained HepG2 cells were incubated with 500 μl Advanced DMEM medium, while as a positive control, the stained HepG2 cells were lysed with 500 μl DMEM+1% Triton X-100. The 24-well plate was then incubated at 37°C for 1 h. Subsequently, the plate was then placed on ice for 20 min to dissolve the trophozoites. After washing twice with NaPBS (4°C), 1 ml of 1% Triton X-100 PBS was pipetted into each well to lyse the HepG2 cells, and the plate was incubated at 37°C for 30 min. 150 μl of the supernatant were pipetted into a black 96-well plate (Greiner, Frickenhausen, Germany), which was centrifuged at 1400 x *g* for 30 sec and measured with a fluorescence plate reader (GENios, TECAN, Fornax Technologies GmbH, Uetikon am See, Switzerland) at an absorbance of 485 nm and an emission of 535 nm. The negative control values were set to 100%. The values of the samples were then related to the negative control. Finally, the result was subtracted from 100% to determine the percentage of cells detached from the cell layer by the trophozoites. Experiments were performed at least 3 times in triplicate.

### Determination of cysteine peptidase activity and substrate gel electrophoresis

Amoebae were seeded in T25 cell culture flasks 24 h prior to the start of the experiment to form a monolayer the next day. To prepare the amoeba extracts used for the determination of cysteine peptidase activity and substrate gel electrophoresis, the amoebae were lysed in liquid nitrogen over 3 freeze-thaw cycles and sedimented at 12,000 x *g* for 15 min at 4°C. The supernatant was used in the corresponding experiments. Protein concentration was measured using the Pierce BCA Protein Assay Kit (Thermo Fisher Scientific) according to the manufacturer’s instructions.

Cysteine peptidase activity was measured in a 96-well plate in a final volume of 200 μl (148 μl CP assay buffer (0.1 M KH_2_PO_4_, 2 mM EDTA, 1 mM DTT), 2 μl amoeba extract and 50 μl of the synthetic peptide Z-Arg-Arg-pNA (400 μM concentration; Bachem, Bubendorf, Switzerland) as substrate. One unit of enzymatic activity is defined as the amount of enzyme that catalyzes the formation of 1 mmol p-nitroaniline in 1 min. For the metallopeptidase transfectants the experiments were performed at least six times in duplicate and the significance was determined using the unpaired *t* test. For the overexpressing transfectant A1^np^HP127^OE^ the experiments were performed four times in duplicate and for the silencing transfectant B2^p^HP127^Si^ the experiments were performed five times in duplicate.

Substrate gel electrophoresis was performed as previously described [4, 53]. Briefly, 4 μg of amoeba extract was separated on a 12% SDS-polyacrylamide gel co-polymerized with 0.1% gelatin. This was followed by incubation with 2.5% Triton X-100 for 1 h, followed by incubation with 100 mM sodium acetate (pH 4.5), 1% Triton X-100, and 20 mM DTT for 3 h at 37°C. Gels were stained with Coomassie to visualize cysteine peptidase activity.

### Determination of hemolytic activity

The hemolytic activity assay was performed as described by Biller *et al*. [3]. For the assay, trophozoites and erythrocytes were mixed at a ratio of 1:2000 (1.25 x 10^5^ amoebae with 2.5 x 10^8^ erythrocytes per ml NaPBS) and then incubated at 37°C for 1 h. After incubation, the cells were sedimented, and the hemoglobin released in the supernatant was measured at 530 nm in a spectrophotometer. Separately incubated erythrocytes and trophozoites were used as negative controls. To determine 100% hemoglobin release, 2.5x10^8^ erythrocytes were lysed in 1 ml of water. The experiments were performed at least three times in quadruplicate for the metallopeptidase transfectants, two times in duplicate for the overexpressing transfectant A1^np^HP127^OE^ and seven times in triplicate for the silencing transfectant B2^p^HP127^Si^.

### Determination of growth rate

To determine growth rate, 500 trophozoites of each amoeba cell line were seeded onto a 24-well plate, and the cells were counted every 24 h over 72 h. Experiments were performed four times in triplicate.

### Statistics

The data are presented as box plots showing the minimum, maximum, median, and all individual measurement points. The data were first checked for normal distribution and homogeneity of variance. In the case of normal distribution, the unpaired t-test was used to compare two groups, and the ordinary one-way ANOVA was used to compare three or more groups. For the ANOVA test, the significance threshold after Bonferroni correction was adjusted to the control level for each comparison. If there was no normal distribution, the Mann-Whitney test was used to compare two groups, and the non-parametric ANOVA test was used for three or more groups. Multiple comparison test was performed according to Benjamini and Hochberg. All statistical analyses were performed using Prism 9, Version 9.3.1 (350), December 7, 2021.

### Western blot

To prepare amoeba extracts for Western blot, trophozoites were washed twice with NaPBS and sedimented by centrifugation at 400 x *g* for 2 min at 4°C. To minimize proteolysis, 20 μM *trans*-Epoxysuccinyl-L-leucylamido(4-guanidino)butan (E64, Sigma-Aldrich, Merck, Taufkirchen, Germany) was added. For lysis the trophozoites were alternately flash frozen in liquid nitrogen for 4 times. The lysates were centrifuged at 40,000 x *g* for 1 h at 4°C. The supernatants contained PBS-soluble proteins. Pellets were washed twice in ice-cold NaPBS and solubilized in NaPBS supplemented with 1% Triton X-100. Extracts (50 μg/lane) were separated on 13% SDS-PAGE gels under reducing conditions. Proteins were transferred to nitrocellulose membranes by the wet blotting technique, with 25 mM Tris-HCl, 192 mM glycine, 1.3 mM SDS, pH 8.3, and 20% methanol as the transfer buffer. For Western blot analysis, a primary anti-c-myc primary antibody (Sigma-Aldrich) at 1:1000 dilution and a secondary anti-mouse horseradish peroxidase (HRP) antibody (Sigma-Aldrich) at 1:5000 dilution was used. Blots were developed using GE Healthcare Amersham ECL Prime Western Blotting Detection Reagent (fisher scientific, Thermo Fisher Scientific, Schwerte, Germany).

### Immunofluorescence analyses

To localize the proteins to be analyzed, transfectants expressing the corresponding c-myc fusion proteins were harvested, washed 1 x with NaPBS, and resuspended in 1 ml NaPBS. After centrifugation at 400 x *g* at 4°C for 5 min, the supernatant was discarded, the cell sediment was resuspended in 1 ml of 4% paraformaldehyde (PFA; Hatfield, PA, USA) in NaPBS, and the trophozoites were incubated for 30 min at RT on a rolling incubator in the NaPBS/4% PFA solution. This and all subsequent incubation steps were performed on a rolling incubator. After centrifugation at 400 x *g* for 3 min, the supernatant was discarded, and the trophozoites were resuspended in 500 μl NaPBS/0.05% saponin (for permeabilization of cell membranes (Sigma-Aldrich, Merck, Taufkirchen, Germany) and incubated for 5 minutes. After centrifugation at 400 x g for 3 minutes, the supernatant was discarded and the trophozoites were resuspended in 500 μl 50 μM ammonium chloride solution to block free aldehyde groups and incubated for an additional 15 min at RT. This was followed by two washing steps with 500 μl NaPBS/0.05% saponin or 500 μl NaPBS (400 x g for 3 min) and an incubation for 10 min with 500 μl NaPBS/2% FCS resuspended. An additional washing step was followed by a 1 h incubation at RT with the primary mouse anti-c-myc antibody (500 μl, 1:100, Sigma Aldrich). Further, three washing steps followed as described above. The cell sediments were then incubated in 500 μl NaPBS containing the secondary fluorescently labeled antibody (1:400, anti-mouse alexa fluor 488, Thermo Fisher Scientific, Bremen, Germany) for another 1 h rolling at RT.

For co-localization, an antibody targeting the cytoplasmic iron-containing superoxide dismutase (SOD; dilution 1:200; [54]) and anti-rabbit Alexa Fluor 594 antibody (dilution 1:400; Thermo Fisher Scientific, Bremen, Germany) and an antibody targeting the α-Gal/GalNAc lectin (dilution 1:200; [55]) and anti-rabbit Alexa Fluor 594 antibody (dilution 1:400) were used. After washing the trophozoites again three times, the nuclei were stained by incubation with Hoechst-33342 (1:400; Invitrogen, Thermo Fisher Scientific, Bremen, Germany) diluted in 500 μl NaPBS for 10 min at RT. After a final washing step the trophozoites were resuspended in 50 μl NaPBS and could be stored in the dark at 4°C until analysis. As a control for each experiment, non-transfected A1^np^ or B2^p^ trophozoites were incubated with the anti-Myc antibody and the secondary fluorescent anti-mouse Alexa Fluor 488-labelled antibody under the same conditions as described for the transfectants. Microscopy was performed using a *Zeiss Axio Imager M2* microscope and an *Olympus FluoView1000* confocal microscope.

## Supporting information

S1 Fig Heatmap of significantly differentially expressed genes (> 1.8 fold, *p*adj < 0.05) in the different *E*. *histolytica* transfectants silencing or overexpressing *ehmp8-1*, *ehmp8-2* or both compared to the corresponding controls. A maximum of 50 genes with the highest fold change are shown. A. A1^np^ versus A1^np^MP8-1^Si^, B. A1^np^ versus A1^np^MP8-2^Si^, C. A1^np^ versus A1^n^pMP8-1+2^Si^, D. B2^p^ versus B2^p^MP8-1^Si^.
(TIF)

S2 Fig Immunofluorescent analysis: Control of the α-myc antibody. To ensure that the α-myc antibody does not lead to a non-specific signal, wild type amoebae of clone B2p were treated with and without saponin and stained with ⍺-c-myc primary antibody (1:200) and α-mouse Alexa Fluor 488 (1:400, green). Nuclei were stained with Hoechst dye (blue).
(TIF)

S3 Fig Heatmap of significantly differentially expressed genes (> 1.8 fold, *p*adj < 0.05) in the different *E*. *histolytica* transfectants silencing or overexpressing *ehmp8-1*, *ehmp8-2* or both compared to the corresponding controls. A maximum of 50 genes with the highest fold change are shown. A. A1^np^ versus A1^np^MP8-1^Si^, B. A1^np^ versus A1^np^MP8-2^Si^, C. A1^np^ versus A1^np^MP8-1+2^Si^, D. B2^p^ versus B2^p^MP8-1^Si^.
(TIF)

S4 Fig Immunofluorescent analysis (IFA): Fixation control. After harvesting and washing, the trophozoites were fixed with 4% paraformaldehyde for 30 min at RT. Half of the trophozoites were then resuspended in 0.05% saponin (for permeabilization of cell membranes) and incubated for 5 minutes and treated with 50 μM ammonium chloride solution to block free aldehyde groups. Trophozoites ±treated with saponin (-Saponin; +Saponin) were then blocked with 2% FCS before incubation for 1 h with the primary mouse α-c-myc antibody (1:100) and the secondary fluorescently labeled antibody (1:400, anti-mouse alexa fluor 488) for another 1 h at RT. For co-localization, an antibody targeting the α-Gal/GalNAc lectin (dilution 1:200; [55]) and anti-rabbit Alexa Fluor 594 antibody (dilution 1:400) were used. Nuclei were stained by incubation with Hoechst-33342 (dilution 1:400). A. IFA analyses of A1^np^MP8-1^Myc^ trophozoites. B. IFA analyses of A1^np^MP8-2^Myc^ trophozoites. C. IFA analyses of A1^np^MP8-2^Myc^ trophozoites; co-localization with surface localized Gal/GalNAc lectin. D IFA analyses of B2^p^EhHP127^Myc^ trophozoites; co-localization with surface localized Gal/GalNAc lectin.
(TIF)

S1 Table RNAseq analyses of clone A1^np^ and A1^np^MP8-1^Si^ silencing transfectant. (XLSX)

S2 Table RNAseq analyses of clone A1^np^ and A1^np^MP8-2^Si^ silencing transfectant. (XLSX)

S3 Table RNAseq analyses of clone A1^np^ and A1^np^MP8-1+2^Si^ silencing transfectant. (XLSX)

S4 Table GO term analyses (GO-BP, GO-MF, GO-CC) of the differentially expressed genes of the comparison between A1^np^ and A1npMP8-1^Si^ silencing transfectant. (XLSX)

S5 Table GO term analyses (GO-BP, GO-MF, GO-CC) of the differentially expressed genes of the comparison between A1^np^ and A1npMP8-2^Si^ silencing transfectant. (XLSX)

S6 Table GO term analyses (GO-BP, GO-MF, GO-CC) of the differentially expressed genes of the comparison between A1^np^ and A1^np^MP8-1+2^Si^ silencing transfectant. (XLSX)

S7 Table RNAseq analyses of clone B2^p^ and B2^p^MP8-1^Si^ silencing transfectant. (XLSX)

S8 Table Comparison of the amino acid sequences of various leishmanolysins and invadolysins with EhMP8-1 and EhMP8-2. Identity in % is shown. (TIF)

S9 Table RNAseq analyses of clone B2^p^ and B2^p^EhHP127^Si^ silencing transfectant. (XLSX)

S10 Table RNAseq analyses of A1^np^pNC and A1^np^HP127^OE^ transfectants. (XLSX)

S11 Table GO term analyses (GO-BP, GO-MF, GO-CC) of the differentially expressed genes of the comparison between A1^np^pNC and A1^np^HP127^OE^ transfectants. (XLSX)

S12 Table Oligonucleotides for qRT-PCR and for generation of pNC-GOI^Myc^. (DOCX)

S1 Prism Data for Fig 1A and 1B. (PZFX)

S2 Prism Data for Fig 5A–5C. (PZFX)

S3 Prism Data for Fig 5D–5F. (PZFX)

S4 Prism Data for Fig 6A–6C. (PZFX)

S5 Prism Data for Fig 6D–6F. (PZFX)

S6 Prism Data for Fig 7A. (PZFX)

S7 Prism Data for Fig 7B and 7C. (PZFX)

S8 Prism Data for Fig 7E and 7F. (PZFX)

S9 Prism Data for Fig 10A and 10B. (PZFX)

S10 Prism Data for Fig 10C and 10D. (PZFX)

S11 Prism Data for Fig 10E and 10F. (PZFX)

S12 Prism Data for Fig 10G and 10H. (PZFX)

S13 Prism Data for Fig 10I and 10J. (PZFX)

## References

[1] Collaborators GBDDD. Estimates of the global, regional, and national morbidity, mortality, and aetiologies of diarrhoea in 195 countries: a systematic analysis for the Global Burden of Disease Study 2016. Lancet Infect Dis. 2018;18(11):1211–28. Epub 2018/09/24. doi: 10.1016/S1473-3099(18)30362-1 ; PubMed Central PMCID: PMC6202444. 30243583 PMC6202444

[2] Biller L, Davis PH, Tillack M, Matthiesen J, Lotter H, Stanley SL Jr., et al. Differences in the transcriptome signatures of two genetically related Entamoeba histolytica cell lines derived from the same isolate with different pathogenic properties. BMC genomics. 2010;11:63. doi: 10.1186/1471-2164-11-63 . 20102605 PMC2823695

[3] Biller L, Schmidt H, Krause E, Gelhaus C, Matthiesen J, Handal G, et al. Comparison of two genetically related Entamoeba histolytica cell lines derived from the same isolate with different pathogenic properties. Proteomics. 2009;9(17):4107–20. doi: 10.1002/pmic.200900022 . 19688750

[4] Meyer M, Fehling H, Matthiesen J, Lorenzen S, Schuldt K, Bernin H, et al. Overexpression of differentially expressed genes identified in non-pathogenic and pathogenic Entamoeba histolytica clones allow identification of new pathogenicity factors involved in amoebic liver abscess formation. PLoS Pathog. 2016;12(8):e1005853. doi: 10.1371/journal.ppat.1005853 ; PubMed Central PMCID: PMC5004846. 27575775 PMC5004846

[5] Matthiesen J, Lender C, Haferkorn A, Fehling H, Meyer M, Matthies T, et al. Trigger-induced RNAi gene silencing to identify pathogenicity factors of Entamoeba histolytica. FASEB J. 2019;33(2):1658–68. doi: 10.1096/fj.201801313R . 30169111

[6] Konig C, Honecker B, Wilson IW, Weedall GD, Hall N, Roeder T, et al. Taxon-Specific Proteins of the Pathogenic Entamoeba Species E. histolytica and E. nuttalli. Front Cell Infect Microbiol. 2021;11:641472. Epub 20210319. doi: 10.3389/fcimb.2021.641472 ; PubMed Central PMCID: PMC8017271. 33816346 PMC8017271

[7] Teixeira JE, Sateriale A, Bessoff KE, Huston CD. Control of Entamoeba histolytica adherence involves metallosurface protease 1, an M8 family surface metalloprotease with homology to leishmanolysin. Infect Immun. 2012;80(6):2165–76. Epub 2012/03/28. doi: 10.1128/IAI.06389-11 ; PubMed Central PMCID: PMC3370588. 22451519 PMC3370588

[8] Rawlings ND, Barrett AJ. Evolutionary families of metallopeptidases. Methods Enzymol. 1995;248:183–228. doi: 10.1016/0076-6879(95)48015-3 . 7674922

[9] Shaulov Y, Sarid L, Trebicz-Geffen M, Ankri S. Entamoeba histolytica Adaption to Auranofin: A Phenotypic and Multi-Omics Characterization. Antioxidants (Basel). 2021;10(8). Epub 20210802. doi: 10.3390/antiox10081240 ; PubMed Central PMCID: PMC8389260. 34439488 PMC8389260

[10] Yanagawa Y, Izumiyama S, Saito-Nakano Y, Nakada-Tsukui K, Kobayashi S, Yoshida N, et al. Gene expression of axenically-isolated clinical Entamoeba histolytica strains and its impact on disease severity of amebiasis. PLoS Pathog. 2022;18(9):e1010880. Epub 20220930. doi: 10.1371/journal.ppat.1010880 ; PubMed Central PMCID: PMC9555656. 36178974 PMC9555656

[11] Yao C, Donelson JE, Wilson ME. The major surface protease (MSP or GP63) of Leishmania sp. Biosynthesis, regulation of expression, and function. Mol Biochem Parasitol. 2003;132(1):1–16. doi: 10.1016/s0166-6851(03)00211-1 . 14563532

[12] Russell DG, Wilhelm H. The involvement of the major surface glycoprotein (gp63) of Leishmania promastigotes in attachment to macrophages. J Immunol. 1986;136(7):2613–20. . 3950420

[13] Puentes SM, Dwyer DM, Bates PA, Joiner KA. Binding and release of C3 from Leishmania donovani promastigotes during incubation in normal human serum. J Immunol. 1989;143(11):3743–9. . 2584716

[14] Connell ND, Medina-Acosta E, McMaster WR, Bloom BR, Russell DG. Effective immunization against cutaneous leishmaniasis with recombinant bacille Calmette-Guerin expressing the Leishmania surface proteinase gp63. Proc Natl Acad Sci U S A. 1993;90(24):11473–7. doi: 10.1073/pnas.90.24.11473 ; PubMed Central PMCID: PMC48006. 8265576 PMC48006

[15] McGwire BS, Chang KP, Engman DM. Migration through the extracellular matrix by the parasitic protozoan Leishmania is enhanced by surface metalloprotease gp63. Infect Immun. 2003;71(2):1008–10. Epub 2003/01/24. doi: 10.1128/IAI.71.2.1008-1010.2003 ; PubMed Central PMCID: PMC145380. 12540585 PMC145380

[16] Soares RP, Altoe ECF, Ennes-Vidal V, da Costa SM, Rangel EF, de Souza NA, et al. In Vitro Inhibition of Leishmania Attachment to Sandfly Midguts and LL-5 Cells by Divalent Metal Chelators, Anti-gp63 and Phosphoglycans. Protist. 2017;168(3):326–34. doi: 10.1016/j.protis.2017.03.004 . 28472733

[17] Rebello KM, Uehara LA, Ennes-Vidal V, Garcia-Gomes AS, Britto C, Azambuja P, et al. Participation of Trypanosoma cruzi gp63 molecules on the interaction with Rhodnius prolixus. Parasitology. 2019:1–8. doi: 10.1017/S0031182019000441 . 31057143 PMC6604109

[18] Grandgenett PM, Otsu K, Wilson HR, Wilson ME, Donelson JE. A function for a specific zinc metalloprotease of African trypanosomes. PLoS Pathog. 2007;3(10):1432–45. doi: 10.1371/journal.ppat.0030150 ; PubMed Central PMCID: PMC2034397. 17953481 PMC2034397

[19] Ma L, Meng Q, Cheng W, Sung Y, Tang P, Hu S, et al. Involvement of the GP63 protease in infection of Trichomonas vaginalis. Parasitol Res. 2011;109(1):71–9. doi: 10.1007/s00436-010-2222-2 . 21221643

[20] de Miguel N, Lustig G, Twu O, Chattopadhyay A, Wohlschlegel JA, Johnson PJ. Proteome analysis of the surface of Trichomonas vaginalis reveals novel proteins and strain-dependent differential expression. Mol Cell Proteomics. 2010;9(7):1554–66. Epub 2010/05/15. doi: 10.1074/mcp.M000022-MCP201 ; PubMed Central PMCID: PMC2938091. 20467041 PMC2938091

[21] Sutter A, Antunes D, Silva-Almeida M, Costa MGS, Caffarena ER. Structural insights into leishmanolysins encoded on chromosome 10 of Leishmania (Viannia) braziliensis. Mem Inst Oswaldo Cruz. 2017;112(9):617–25. doi: 10.1590/0074-02760160522 ; PubMed Central PMCID: PMC5572447. 28902287 PMC5572447

[22] McHugh B, Krause SA, Yu B, Deans AM, Heasman S, McLaughlin P, et al. Invadolysin: a novel, conserved metalloprotease links mitotic structural rearrangements with cell migration. J Cell Biol. 2004;167(4):673–86. doi: 10.1083/jcb.200405155 ; PubMed Central PMCID: PMC2172566. 15557119 PMC2172566

[23] Cobbe N, Marshall KM, Gururaja Rao S, Chang CW, Di Cara F, Duca E, et al. The conserved metalloprotease invadolysin localizes to the surface of lipid droplets. J Cell Sci. 2009;122(Pt 18):3414–23. Epub 20090825. doi: 10.1242/jcs.044610 ; PubMed Central PMCID: PMC2736869. 19706689 PMC2736869

[24] Ayala I, Baldassarre M, Caldieri G, Buccione R. Invadopodia: a guided tour. Eur J Cell Biol. 2006;85(3–4):159–64. Epub 20051011. doi: 10.1016/j.ejcb.2005.09.005 . 16546558

[25] Grande-Garcia A, del Pozo MA. Caveolin-1 in cell polarization and directional migration. Eur J Cell Biol. 2008;87(8–9):641–7. Epub 20080328. doi: 10.1016/j.ejcb.2008.02.001 . 18375013

[26] Rappoport JZ, Simon SM. Real-time analysis of clathrin-mediated endocytosis during cell migration. J Cell Sci. 2003;116(Pt 5):847–55. doi: 10.1242/jcs.00289 . 12571282

[27] Hasan MM, Teixeira JE, Huston CD. Invadosome-Mediated Human Extracellular Matrix Degradation by Entamoeba histolytica. Infect Immun. 2018;86(9). Epub 20180822. doi: 10.1128/IAI.00287-18 ; PubMed Central PMCID: PMC6105895. 29914929 PMC6105895

[28] Freiburghaus AU, Roduner J, Hadorn HB. Activation of Human Pancreatic Proteolytic Enzymes: The Role of Enteropeptidase and Trypsin. JPGN Reports. 2021;2(4):e138. doi: 10.1097/PG9.0000000000000138 -202111000-00021. 37206452 PMC10191478

[29] Shi Y. Caspase activation, inhibition, and reactivation: A mechanistic view. Protein Science. 2004;13(8):1979–87. doi: 10.1110/ps.04789804 15273300 PMC2279816

[30] Reuber TL, Ausubel FM. Isolation of Arabidopsis genes that differentiate between resistance responses mediated by the RPS2 and RPM1 disease resistance genes. Plant Cell. 1996;8(2):241–9. doi: 10.1105/tpc.8.2.241 ; PubMed Central PMCID: PMC161094. 8742710 PMC161094

[31] Nitta T, Takahama Y. The lymphocyte guard-IANs: regulation of lymphocyte survival by IAN/GIMAP family proteins. Trends Immunol. 2007;28(2):58–65. Epub 20061228. doi: 10.1016/j.it.2006.12.002 . 17196432

[32] Nakada-Tsukui K, Sekizuka T, Sato-Ebine E, Escueta-de Cadiz A, Ji DD, Tomii K, et al. AIG1 affects in vitro and in vivo virulence in clinical isolates of Entamoeba histolytica. PLoS Pathog. 2018;14(3):e1006882. Epub 20180319. doi: 10.1371/journal.ppat.1006882 ; PubMed Central PMCID: PMC5884625. 29554130 PMC5884625

[33] Lozano-Mendoza J, Ramirez-Montiel F, Rangel-Serrano A, Paramo-Perez I, Mendoza-Macias CL, Saavedra-Salazar F, et al. Attenuation of In Vitro and In Vivo Virulence Is Associated with Repression of Gene Expression of AIG1 Gene in Entamoeba histolytica. Pathogens. 2023;12(3). Epub 20230321. doi: 10.3390/pathogens12030489 ; PubMed Central PMCID: PMC10051847. 36986411 PMC10051847

[34] Vicente JB, Ehrenkaufer GM, Saraiva LM, Teixeira M, Singh U. Entamoeba histolytica modulates a complex repertoire of novel genes in response to oxidative and nitrosative stresses: implications for amebic pathogenesis. Cell Microbiol. 2009;11(1):51–69. Epub 20080905. doi: 10.1111/j.1462-5822.2008.01236.x ; PubMed Central PMCID: PMC3418052. 18778413 PMC3418052

[35] Penuliar GM, Nakada-Tsukui K, Nozaki T. Phenotypic and transcriptional profiling in Entamoeba histolytica reveal costs to fitness and adaptive responses associated with metronidazole resistance. Front Microbiol. 2015;6:354. Epub 20150505. doi: 10.3389/fmicb.2015.00354 ; PubMed Central PMCID: PMC4419850. 25999919 PMC4419850

[36] Gilchrist CA, Houpt E, Trapaidze N, Fei Z, Crasta O, Asgharpour A, et al. Impact of intestinal colonization and invasion on the Entamoeba histolytica transcriptome. Mol Biochem Parasitol. 2006;147(2):163–76. Epub 20060307. doi: 10.1016/j.molbiopara.2006.02.007 . 16569449

[37] Weber C, Koutero M, Dillies MA, Varet H, Lopez-Camarillo C, Coppee JY, et al. Extensive transcriptome analysis correlates the plasticity of Entamoeba histolytica pathogenesis to rapid phenotype changes depending on the environment. Sci Rep. 2016;6:35852. Epub 20161021. doi: 10.1038/srep35852 ; PubMed Central PMCID: PMC5073345. 27767091 PMC5073345

[38] Ehrenkaufer GM, Haque R, Hackney JA, Eichinger DJ, Singh U. Identification of developmentally regulated genes in Entamoeba histolytica: insights into mechanisms of stage conversion in a protozoan parasite. Cell Microbiol. 2007;9(6):1426–44. Epub 20070122. doi: 10.1111/j.1462-5822.2006.00882.x . 17250591

[39] Irmer H, Tillack M, Biller L, Handal G, Leippe M, Roeder T, et al. Major cysteine peptidases of Entamoeba histolytica are required for aggregation and digestion of erythrocytes but are dispensable for phagocytosis and cytopathogenicity. Mol Microbiol. 2009;72(3):658–67. doi: 10.1111/j.1365-2958.2009.06672.x . 19426210

[40] Ankri S, Stolarsky T, Mirelman D. Antisense inhibition of expression of cysteine proteinases does not affect Entamoeba histolytica cytopathic or haemolytic activity but inhibits phagocytosis. Mol Microbiol. 1998;28(4):777–85. doi: 10.1046/j.1365-2958.1998.00837.x . 9643545

[41] Jacobs T, Bruchhaus I, Dandekar T, Tannich E, Leippe M. Isolation and molecular characterization of a surface-bound proteinase of Entamoeba histolytica. Mol Microbiol. 1998;27(2):269–76. doi: 10.1046/j.1365-2958.1998.00662.x . 9484883

[42] Melendez-Lopez SG, Herdman S, Hirata K, Choi MH, Choe Y, Craik C, et al. Use of recombinant Entamoeba histolytica cysteine proteinase 1 to identify a potent inhibitor of amebic invasion in a human colonic model. Eukaryot Cell. 2007;6(7):1130–6. Epub 20070518. doi: 10.1128/EC.00094-07 ; PubMed Central PMCID: PMC1951106. 17513563 PMC1951106

[43] Riekenberg S, Witjes B, Saric M, Bruchhaus I, Scholze H. Identification of EhICP1, a chagasin-like cysteine protease inhibitor of Entamoeba histolytica. FEBS Lett. 2005;579(7):1573–8. doi: 10.1016/j.febslet.2005.01.067 . 15757643

[44] Saric M, Vahrmann A, Bruchhaus I, Bakker-Grunwald T, Scholze H. The second cysteine protease inhibitor, EhICP2, has a different localization in trophozoites of Entamoeba histolytica than EhICP1. Parasitol Res. 2006;100(1):171–4. Epub 20060627. doi: 10.1007/s00436-006-0206-z . 16802137

[45] Okada M, Huston CD, Mann BJ, Petri WA Jr., Kita K, Nozaki T. Proteomic analysis of phagocytosis in the enteric protozoan parasite Entamoeba histolytica. Eukaryot Cell. 2005;4(4):827–31. doi: 10.1128/EC.4.4.827-831.2005 ; PubMed Central PMCID: PMC1087816. 15821141 PMC1087816

[46] Que X, Brinen LS, Perkins P, Herdman S, Hirata K, Torian BE, et al. Cysteine proteinases from distinct cellular compartments are recruited to phagocytic vesicles by Entamoeba histolytica. Mol Biochem Parasitol. 2002;119(1):23–32. doi: 10.1016/s0166-6851(01)00387-5 . 11755183

[47] Ocadiz-Ruiz R, Fonseca W, Linford AS, Yoshino TP, Orozco E, Rodriguez MA. The knockdown of each component of the cysteine proteinase-adhesin complex of Entamoeba histolytica (EhCPADH) affects the expression of the other complex element as well as the in vitro and in vivo virulence. Parasitology. 2016;143(1):50–9. Epub 20151102. doi: 10.1017/S003118201500147X . 26521708

[48] Diamond LS, Harlow DR, Cunnick CC. A new medium for the axenic cultivation of Entamoeba histolytica and other Entamoeba. Trans R Soc Trop Med Hyg. 1978;72(4):431–2. doi: 10.1016/0035-9203(78)90144-x 212851

[49] Bolger AM, Lohse M, Usadel B. Trimmomatic: a flexible trimmer for Illumina sequence data. Bioinformatics. 2014;30(15):2114–20. doi: 10.1093/bioinformatics/btu170 ; PubMed Central PMCID: PMC4103590. 24695404 PMC4103590

[50] Li B, Dewey CN. RSEM: accurate transcript quantification from RNA-Seq data with or without a reference genome. BMC Bioinformatics. 2011;12:323. doi: 10.1186/1471-2105-12-323 ; PubMed Central PMCID: PMC3163565. 21816040 PMC3163565

[51] Langmead B, Salzberg SL. Fast gapped-read alignment with Bowtie 2. Nat Methods. 2012;9(4):357–9. Epub 2012/03/06. doi: 10.1038/nmeth.1923 ; PubMed Central PMCID: PMC3322381. 22388286 PMC3322381

[52] Love MI, Huber W, Anders S. Moderated estimation of fold change and dispersion for RNA-seq data with DESeq2. Genome Biol. 2014;15(12):550. Epub 2014/12/18. doi: 10.1186/s13059-014-0550-8 ; PubMed Central PMCID: PMC4302049. 25516281 PMC4302049

[53] Hellberg A, Leippe M, Bruchhaus I. Two major ’higher molecular mass proteinases’ of Entamoeba histolytica are identified as cysteine proteinases 1 and 2. Mol Biochem Parasitol. 2000;105(2):305–9. doi: 10.1016/s0166-6851(99)00194-2 . 10693753

[54] Bruchhaus I, Brattig NW, Tannich E. Recombinant expression, purification and biochemical characterization of a superoxide dismutase from Entamoeba histolytica. Arch Med Res. 1992;23(2):27–9. . 1340312

[55] Biller L, Matthiesen J, Kuhne V, Lotter H, Handal G, Nozaki T, et al. The cell surface proteome of Entamoeba histolytica. Mol Cell Proteomics. 2014;13(1):132–44. Epub 20131017. doi: 10.1074/mcp.M113.031393 ; PubMed Central PMCID: PMC3879609. 24136294 PMC3879609

