# Supplementary figures and images for: Genes differentially expressed between pathogenic and non-pathogenic Entamoeba histolytica clones influence pathogenicity-associated phenotypes by multiple mechanisms
Source: PLoS Pathog. 2023 Dec 22;19(12):e1011745. doi: 10.1371/journal.ppat.1011745 (PMC10773965; doi:10.1371/journal.ppat.1011745)

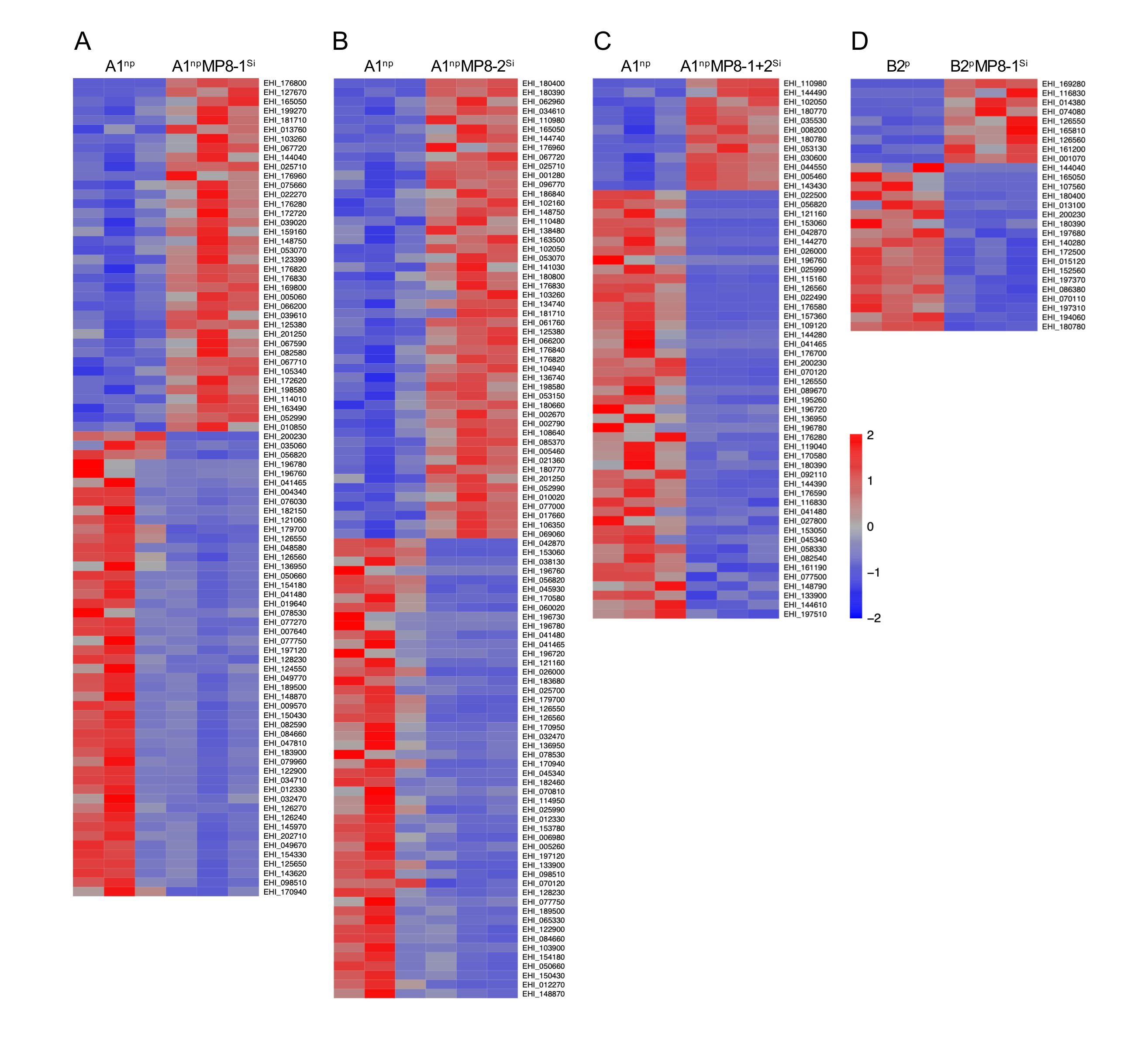

Supplement: S1 Fig — A maximum of 50 genes with the highest fold change are shown. A. A1np versus A1npMP8-1Si, B. A1np versus A1npMP8-2Si, C. A1np versus A1npMP8-1+2Si, D. B2p versus B2pMP8-1Si. (TIF) [file ppat.1011745.s001.tif]

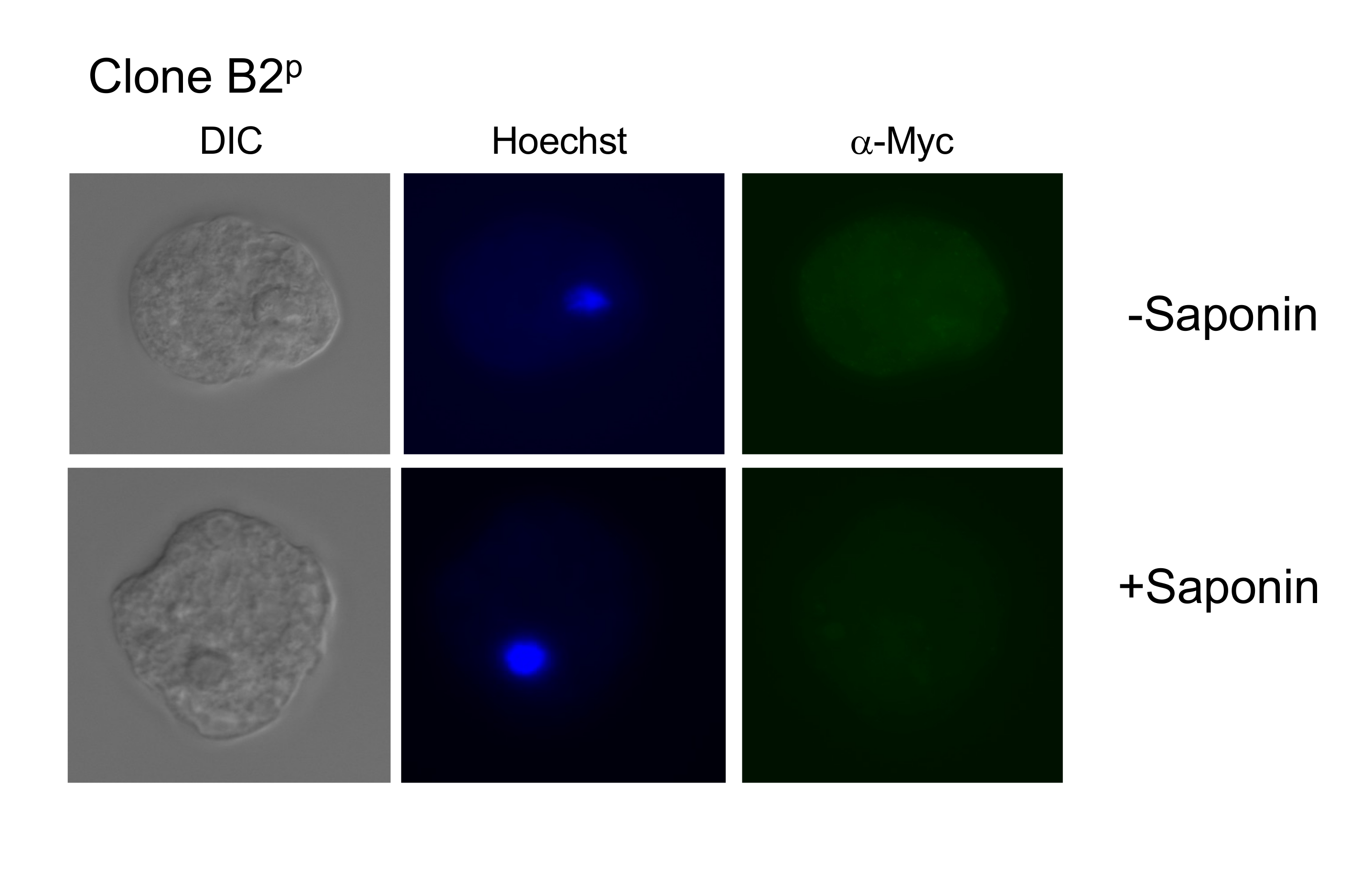

Supplement: S2 Fig — To ensure that the α-myc antibody does not lead to a non-specific signal, wild type amoebae of clone B2p were treated with and without saponin and stained with ⍺-c-myc primary antibody (1:200) and α-mouse Alexa Fluor 488 (1:400, green). Nuclei were stained with Hoechst dye (blue). (TIF) [file ppat.1011745.s002.tif]

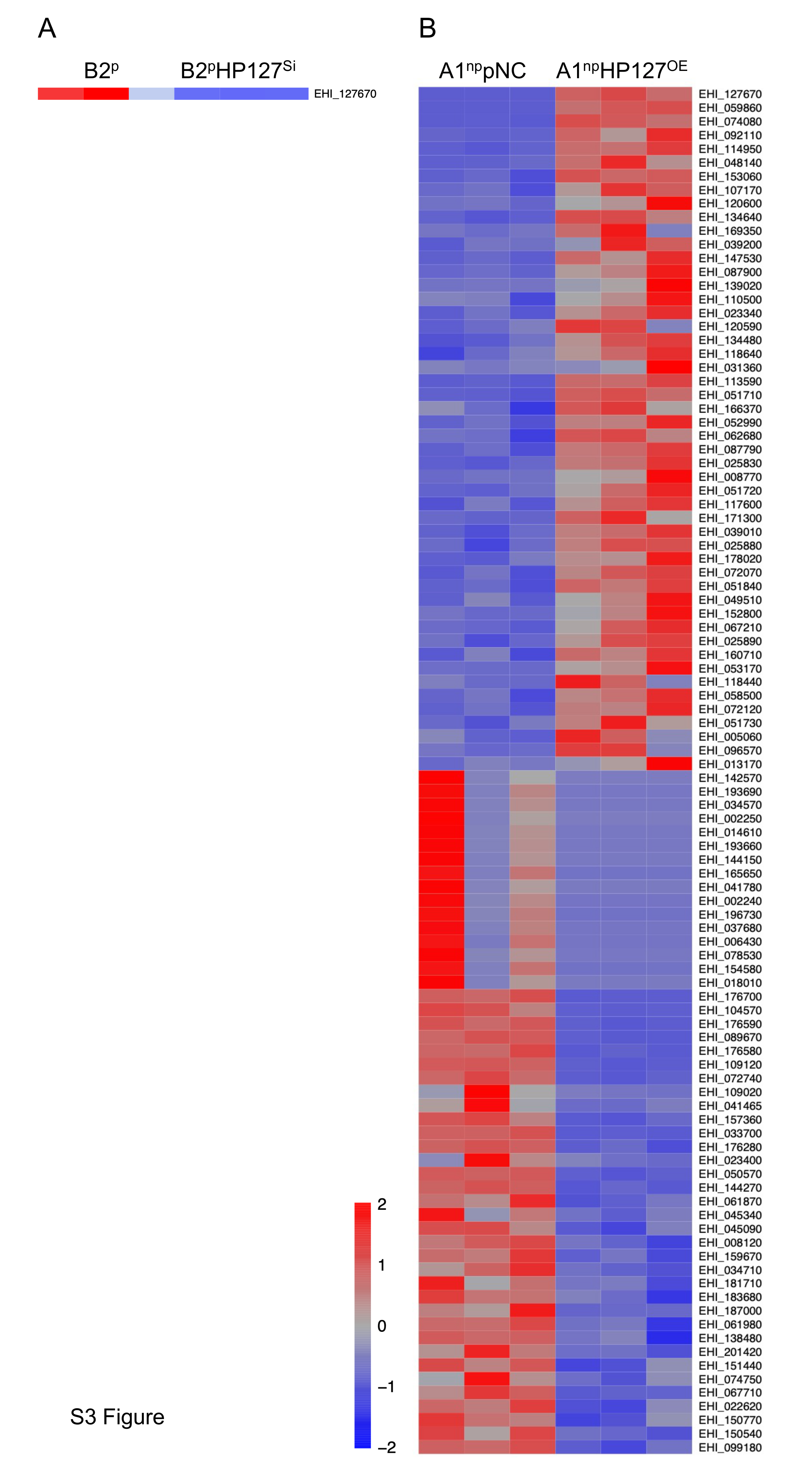

Supplement: S3 Fig — A maximum of 50 genes with the highest fold change are shown. A. A1np versus A1npMP8-1Si, B. A1np versus A1npMP8-2Si, C. A1np versus A1npMP8-1+2Si, D. B2p versus B2pMP8-1Si. (TIF) [file ppat.1011745.s003.tif]

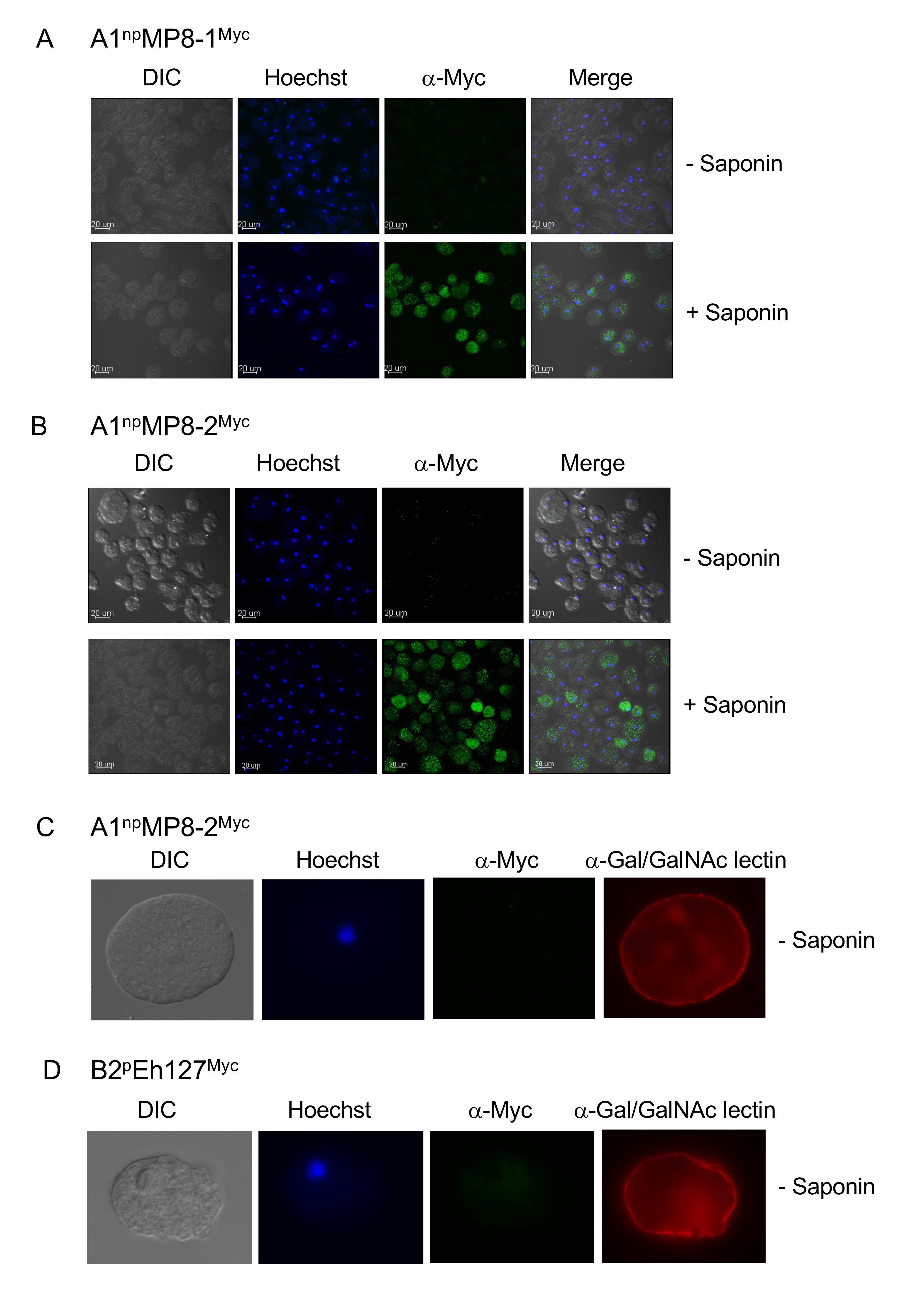

Supplement: S4 Fig — After harvesting and washing, the trophozoites were fixed with 4% paraformaldehyde for 30 min at RT. Half of the trophozoites were then resuspended in 0.05% saponin (for permeabilization of cell membranes) and incubated for 5 minutes and treated with 50 μM ammonium chloride solution to block free aldehyde groups. Trophozoites ±treated with saponin (-Saponin; +Saponin) were then blocked with 2% FCS before incubation for 1 h with the primary mouse α-c-myc antibody (1:100) and the secondary fluorescently labeled antibody (1:400, anti-mouse alexa fluor 488) for another 1 h at RT. For co-localization, an antibody targeting the α-Gal/GalNAc lectin (dilution 1:200; [55]) and anti-rabbit Alexa Fluor 594 antibody (dilution 1:400) were used. Nuclei were stained by incubation with Hoechst-33342 (dilution 1:400). A. IFA analyses of A1npMP8-1Myc trophozoites. B. IFA analyses of A1npMP8-2Myc trophozoites. C. IFA analyses of A1npMP8-2Myc trophozoites; co-localization with surface localized Gal/GalNAc lectin. D IFA analyses of B2pEhHP127Myc trophozoites; co-localization with surface localized Gal/GalNAc lectin. (TIF) [file ppat.1011745.s004.tif]

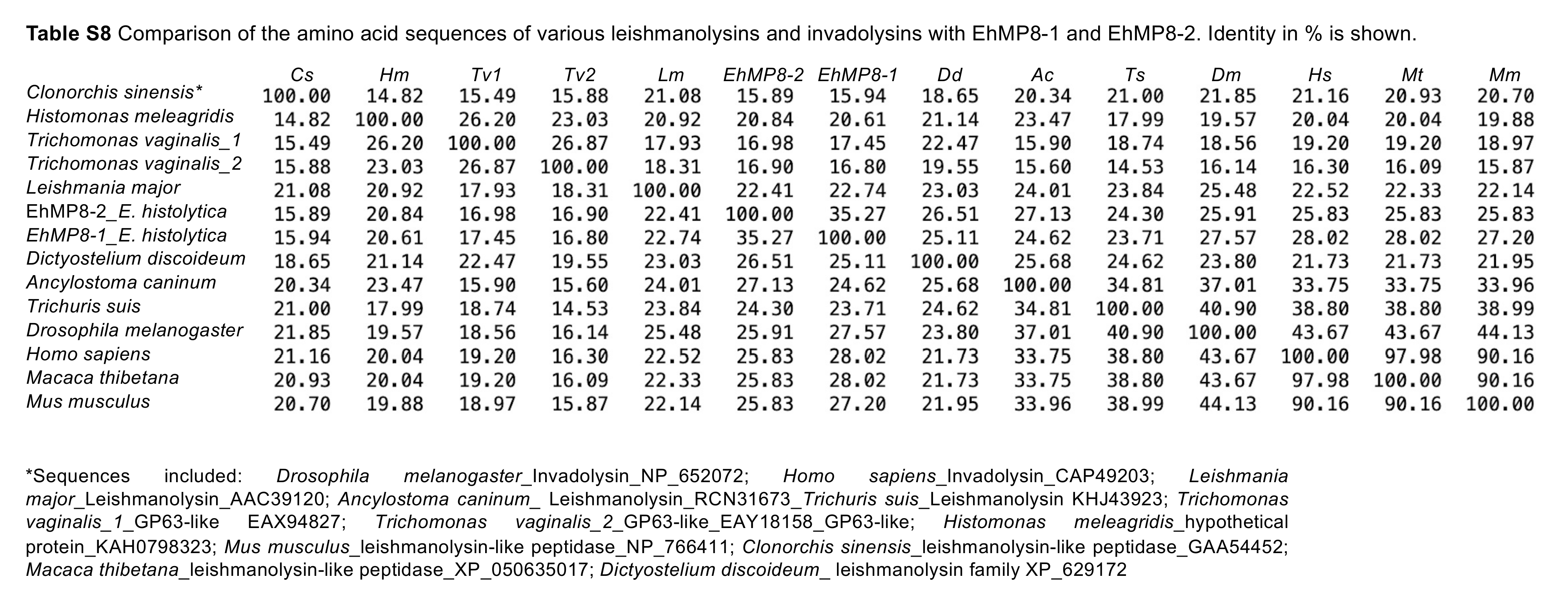

Supplement: S8 Table — (TIF) [file ppat.1011745.s012.tif]
