# Supplementary material for: Genes differentially expressed between pathogenic and non-pathogenic Entamoeba histolytica clones influence pathogenicity-associated phenotypes by multiple mechanisms
Source: PLoS Pathog. 2023 Dec 22;19(12):e1011745. doi: 10.1371/journal.ppat.1011745 (PMC10773965; doi:10.1371/journal.ppat.1011745)
Supplement: S12 Table — (DOCX) [file ppat.1011745.s016.docx]

**Table S12** Oligonucleotides for qRT-PCR and for generation of pNC-GOI^Myc^

| qRT-PCR | | |
| --- | --- | --- |
| Gene, | Sense primer | Antisense primer |
| *ehmp8-1*  (*EHI_200230*) | CAATGCCCATACTGTCCTTC | CTGTCTCTGTTCCTCTTCCT |
| *ehmp8-2* (*EHI_042870*) | ATTGCTGTCATTCCTGTGTG | TCACGAACTTCTTCTGCTTG |
| *ehhp127* (*EHI_127670*) | AGAGAGCTACTCAACATGG | CATATTCATCACACTCTTCC |
| *ehact* (*EHI_142730*) | AAGCTGCATCAAGCAGTGAA | GGAATGATGGTTGGAAGAGG |
| Synthesis of *c-myc* tag | | |
| *c-myc* | CGCTAGCGGATCCGAACAAAAATTAATTTCAGAAGAAGATCTTCTCGAGTAAA | GATCTTTACTCGAGAAGATCTTCTTCTGAAATTAATTTTTGTTCGGATCCGCTAGCGGTAC |
| Amplification of *ehhp127* for generation of pNCEhHP127^Myc^ | | |
| *ehhp127* (*EHI_127670*) | GAGAGGTACCGAATA AATGGAGTAAATAAG | GAGAGGATCCGTAT AAGCAAGTAACAAT ATGTTC |
| Amplification of *ehmp8-1* and *EhMP8-2* for generation of pNCMP8-1^Myc^ and pNCMP8-1^Myc^ | | |
| *ehmp8-1* (*EHI_042870*) | GAGA GGTACC CAAAACTTGTATTTATTG | GAGA GGATCC AACAAGATTTCTTGC |
| *ehmp8-2*  (*EHI_200230*) | GAGA GGTACC GAGTATTAAATGCACTTT | GAGA GGATCC AAACATAACACAAAAGC |
|  |  |  |
|  |  |  |
